# A comprehensive epigenome map of *Plasmodium falciparum* reveals unique mechanisms of transcriptional regulation and identifies H3K36me2 as a global mark of gene suppression

**DOI:** 10.1186/s13072-015-0029-1

**Published:** 2015-09-17

**Authors:** Krishanpal Karmodiya, Saurabh J. Pradhan, Bhagyashree Joshi, Rahul Jangid, Puli Chandramouli Reddy, Sanjeev Galande

**Affiliations:** Department of Biology, Indian Institute of Science Education and Research, Pune, Maharashtra India; Centre of Excellence in Epigenetics, Indian Institute of Science Education and Research, Pune, India; National Centre for Cell Science, Pune, India

**Keywords:** Genome-wide mapping, Histone modifications, Chromatin, Transcription, *Plasmodium*, Virulence and pathogenicity genes

## Abstract

**Background:**

Role of epigenetic mechanisms towards regulation of the complex life cycle/pathogenesis of *Plasmodium falciparum*, the causative agent of malaria, has been poorly understood. To elucidate stage-specific epigenetic regulation, we performed genome-wide mapping of multiple histone modifications of *P. falciparum*. Further to understand the differences in transcription regulation in *P. falciparum* and its host, human, we compared their histone modification profiles.

**Results:**

Our comprehensive comparative analysis suggests distinct mode of transcriptional regulation in malaria parasite by virtue of poised genes and differential histone modifications. Furthermore, analysis of histone modification profiles predicted 562 genes producing anti-sense RNAs and 335 genes having bidirectional promoter activity, which raises the intriguing possibility of RNA-mediated regulation of transcription in *P. falciparum.* Interestingly, we found that H3K36me2 acts as a global repressive mark and gene regulation is fine tuned by the ratio of activation marks to H3K36me2 in *P. falciparum*. This novel mechanism of gene regulation is supported by the fact that knockout of SET genes (responsible for H3K36 methylation) leads to up-regulation of genes with highest occupancy of H3K36me2 in wild-type *P. falciparum*. Moreover, *virulence (var)* genes are mostly poised and marked by a unique set of activation (H4ac) and repression (H3K9me3) marks, which are mutually exclusive to other *Plasmodium* housekeeping genes.

**Conclusions:**

Our study reveals unique plasticity in the epigenetic regulation in *P. falciparum* which can influence parasite virulence and pathogenicity. The observed differences in the histone code and transcriptional regulation in *P. falciparum* and its host will open new avenues for epigenetic drug development against malaria parasite.

**Electronic supplementary material:**

The online version of this article (doi:10.1186/s13072-015-0029-1) contains supplementary material, which is available to authorized users.

## Background

Malaria is a major public health problem in many developing countries, with the parasite *Plasmodium falciparum* causing most of the malaria-associated mortality. *P. falciparum* exhibits a complex life cycle progressing through multiple developmental stages in two hosts. The clinical manifestation of malaria is a result of parasite development in red blood cells (RBCs), where it completes its asexual intra-erythrocytic cycle (IEC). During the 48-h IEC, parasite invades RBC and develops into a ring stage, followed by trophozoite and schizont stages. Nuclear division during the schizont stage results in the formation of 16–32 merozoites, which can infect the new RBCs. To sustain chronic infection in human hosts, parasite undergoes rapid transitions between morphological states, a mechanism of immune evasion that contributes to pathogenicity. These rapid transitions between morphological states are orchestrated by multiple types of transcriptional and epigenetic regulations [1, 2].

Nucleosome is the fundamental unit of chromatin, in which 147 base pairs of DNA are wrapped around histone octamer consisting of two copies each of the four core histone proteins H3, H4, H2A and H2B [3]. Not surprisingly, *P. falciparum* genome encodes the four conserved core histones [4], and its nuclear genome assumes the nucleosomal organization typical of eukaryotes [5]. The N-terminal of core histones protruding from the nucleosome particle is subjected to a variety of post-translational modifications that can modulate gene expression [6]. Extensive studies in multiple model organisms have established that histone acetylation is primarily associated with gene activation whereas methylation is associated with repression and activation depending on its position and state [7]. The levels of acetylation and methylation are regulated by the activity of histone acetyl transferases (HATs) or histone deacetylases (HDACs) and histone methyltransferases (HMTs) or histone demethylases (HDMs), respectively. Multiple studies have suggested critical roles of HDACs and HMTs in controlling gene expression in *P. falciparum* [8–10]. Importantly, majority of *P. falciparum* genes are activated only once during the infected RBC cycle attesting the importance of stringent gene regulation in stage-specific manner [11, 12]. Epigenetic mechanisms have been implicated in regulation of genes playing role in parasite virulence, differentiation and cell-cycle control [13].

Post-translational modifications of histones influence gene expression which can be decoded to decipher the function of underlying DNA sequence. Unlike higher eukaryotes, but similar to *Saccharomyces cerevisiae* and *Tetrahymena thermophila*, a large proportion of the *P. falciparum* genome is constitutively acetylated [14, 15]. Surprisingly, activation marks H3K9ac and H3K4me3 are mainly shown to be located in intergenic regions in *P. falciparum* [16]. In contrast, the typically repressive mark H3K9me3 is exclusively found on virulence gene clusters [16]. However, because of lack of promoter characterization and comprehensive integrative analysis of histone modifications in *P. falciparum*, the extent to which gene-specific combinatorial patterns exist remains to be determined. In this study, by characterizing promoters and analyzing histone modifications at three different IEC developmental stages of *P. falciparum*; ring, trophozoite and schizont, we have unraveled the epigenetic landscape associated with gene regulation. We show that activation marks H3K4me2, H3K4me3, H3K9ac, H3K14ac and histone H4ac are located at the promoter and body of the genes like other eukaryotes and contrary to the earlier observations in *P. falciparum* [16]. We also provide evidence that H3K36me2 acts as a global repressive mark in *P. falciparum* and gene expression is governed by the ratio of activation marks to H3K36me2. Furthermore, relevance of this epigenomic landscape is highlighted by the integration of RNA sequencing, anti-sense transcripts [17] and gene expression profiling dataset for knockout conditions of HMTs (SET domain containing family) in *P. falciparum* [8]. Thus, our integrative analysis reveals important insights into the dynamic as well as static components of the malaria epigenome and provides wealth of information that will be instrumental towards dissecting the molecular events during IEC of *P. falciparum.*

## Results

### Generation of comprehensive epigenomic reference maps for three different intra-erythrocytic stages of *P. falciparum*

**Fig. 1:**
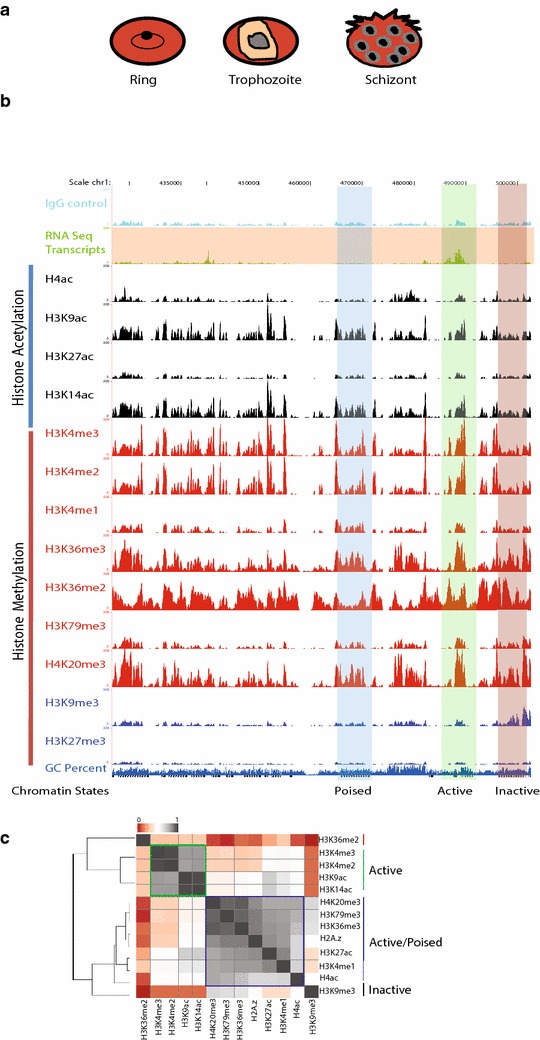
Generation of comprehensive epigenomic maps of histone modifications at three different stages of intra-erythrocytic stages of *P. falciparum* life cycle. **a** Schematic representation of different stages of *Plasmodium falciparum* harvested at 18, 30 and 40 h post-synchronization. A single large-scale culture of highly synchronized cells was used for ChIP-sequencing of all histone modifications. **b** A snapshot of the representative genome browser shows the histone methylation and acetylation, and RNA sequencing reads at trophozoite stage. Representative active chromatin (presence of RNA-seq reads), inactive chromatin (presence of H3K9me3 and absence of RNA-seq reads) and poised chromatin (present of histone activation marks but no RNA-seq reads) are highlighted in* green, red* and* blue color*, respectively. Anti-IgG antibody was used as a negative control. **c** Heat map representing the correlation between occupancy of indicated histone modifications in the gene body for all *P. falciparum* genes. Active promoter marks; H3K4me2, H3K4me3, H3K9ac and H3K14ac clustered separately to form an active chromatin state (*green square*). Histone variant H2A.z and other histone modifications; H4K20me3, H3K79me3, H3K36me3, H3K27ac, H3K4me1 and H4ac are clustered together to form active/poised chromatin state (*blue square*). H3K9me3 and H3K36me2 are anti-correlated with each other suggesting mutually exclusive function for these two histone modifications

To gain insights into the epigenomic landscape of *P. falciparum*, we performed chromatin immunoprecipitation (ChIP)-coupled high-throughput sequencing (ChIP-seq) of active (H3K4me2, H3K4me3, H3K9ac, H3K14ac, H3K27ac and H4ac), inactive (H3K9me3 and H3K27me3), elongation (H3K79me3) and regulatory element (H3K4me1) histone modifications at three different stages of intra-erythrocytic cycle (Additional file 1: Table S1). A large-scale culture of highly synchronized cells was grown, and samples were collected at 18, 30 and 40 h for ring, trophozoite, and schizont stages of *P. falciparum* IEC (Fig. 1a). Moreover, selected histone modification peaks were also validated by ChIP-qPCR (Additional file 1: Figure S1A). Further to confirm if H3K9ac and H3K4me3 co-occupy these loci or it is an effect of cellular heterogeneity, we performed sequential ChIP for H3K9ac followed by H3K4me3 (Additional file 1: Figure S1B). Sequential ChIP demonstrates that given genomic loci have both H3K9ac and H3K4me3 modifications simultaneously. For all three stages and each histone modification, we obtained average transcribed genome coverage of ~50× (Additional file 1: Figure S2). Next we compared our ChIP-seq data with the publicly available data for H3K4me3 and H3K9me3 [16] occupancy in *P. falciparum.* Our data exhibited Pearson correlation coefficient 0.91 and 0.88 for H3K4me3 and H3K9me3, respectively, with publicly available data sets [16] (Additional file 1: Figure S3; and identical profile for H3K4me3, Additional file 1: Figure S4) suggesting significant correlation between them. Further, to generate comprehensive epigenomic map, we have integrated ChIP-seq data for histone variant (H2A.z) and modifications (H3K36me2, H3K36me3 and H4K20me3) and RNA sequencing data available for *P. falciparum* [8, 18] (Fig. 1b, Additional file 1: Table S2).

Our comprehensive analysis of histone modifications suggests that most of the genes (approximately 80 %, RPKM >10) in *P. falciparum* genome are either active or poised. We have defined poised genes as those exhibiting high levels of histone activation marks but no transcription, as evidenced by representative example from the trophozoite stage (Fig. 1b). Surprisingly, analysis of the occupancy of histone modifications revealed that even transcriptionally silent chromatin, based on absence of RNA sequencing reads and presence of H3K9me3, exhibited co-occupancy of active histone modifications (Fig. 1b). Not surprisingly, we could not detect the repression-associated modification H3K27me3 in our ChIP-seq data (Fig. 1b) as the presence of H3K27me3 has not been detected in *Plasmodium* until now [19]. To understand the correlative behavior of histone modifications with their occupancy, we calculated Pearson correlation coefficient in the gene body of all 5265 *P. falciparum* genes. A heat map representation of various histone modifications suggests that active histone modifications (H3K4me2, H3K4me3, H3K9ac and H3K14ac) are grouped together indicating a cumulative action presumably for efficient gene expression (Fig. 1c, green square). Other histone modifications (H4ac, H4K20me3, H3K79me3, H3K27ac, H3K36me3, H3K4me1) and histone variant H2A.z are grouped together indicating that these modifications and histone variant H2A.z along with active histone modifications mark the active and poised chromatin in *P. falciparum* (Fig. 1c, blue square).

### Comparison of histone modification profiles in *P. falciparum* and human cell line

**Fig. 2:**
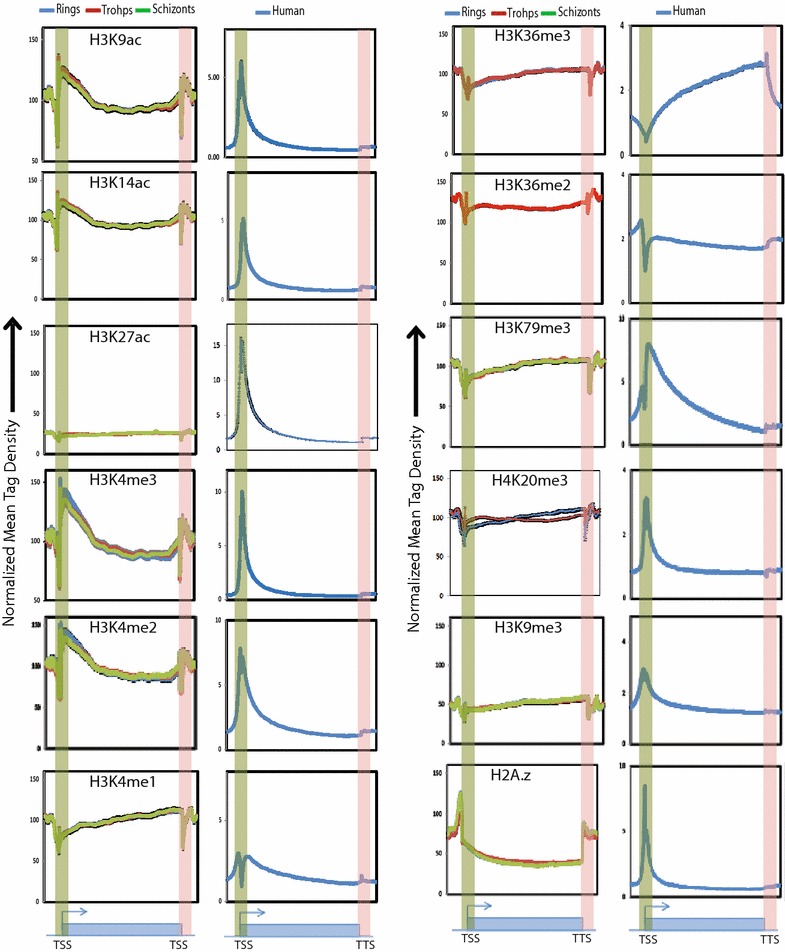
Comparison of histone modification profiles of *P. falciparum* and human genes. Normalized mean tag density gene profile of histone modifications over 5265 *P. falciparum* and 41,390 human genes. Transcription start site (TSS) and transcription termination site (TTS) are highlighted in *green* and *red color*, respectively. *Error bars* (*black*) standard error of the mean. Multiple histone modifications exhibit differential profiles in *P. falciparum* than in human cells suggesting differential mode of gene expression in the malaria parasite

To understand the mechanism(s) of differential transcriptional regulation in *P. falciparum* and its host *Homo sapiens*, we first interrogated how the histone modifications are represented between them. Towards this, first we characterized promoters in *P. falciparum* using the RNA sequencing data from different stages [18]. Next, we examined the distribution profiles of histone modifications and during different stages of *P. falciparum* IEC and compared it with profiles of histone modifications in human (Additional file 1: Table S3). The characteristic promoter-associated peaks for multiple histone activation marks (H3K9ac, H3K14ac, H3K4me2, and H3K4me3) were observed in *Plasmodium*. We also observed that levels of the activation mark H3K27ac were very low as compared to other histone modifications. The profiles of histone modifications H3K36me2 and H3K36me3 in *P. falciparum*, which are associated with transcription elongation in human, match closely with that of humans profile (Fig. 2). H3K27me3, a global repression mark in human, was not detected at all in *P. falciparum* by ChIP-sequencing (data not shown). Another major repression mark in human, H3K9me3, exhibits weak global profile in *P. falciparum* as it is present only on a subset of genes corroborating the earlier findings [16, 20]. Incorporation of the histone variant H2A.z is a mark of active promoters in human and mouse system [21]. However, in *Plasmodium*, H2A.z was present neither at the promoters nor in the gene body. H2A.z occupancy indeed was found flanking the gene unit suggesting that it acts differently in *Plasmodium* presumably as a guard or marker of transcription unit, as suggested earlier [18, 22]. H3K4me1 (mark of enhancers) and H4K20me3 (mark of heterochromatin regions) also showed a differential profile than the human system, though further studies need to be done to understand their function in *P. falciparum*. Thus, the absence of repressive mark H3K27me3 and presence of H3K9me3 only on a subset of genes in *P. falciparum* suggest differential mode of gene regulation in the malaria parasite as compared to its host, human.

### Active histone modifications positively correlate with transcription and H3K36me2 is a potential global repressive mark in *P. falciparum*

**Fig. 3:**
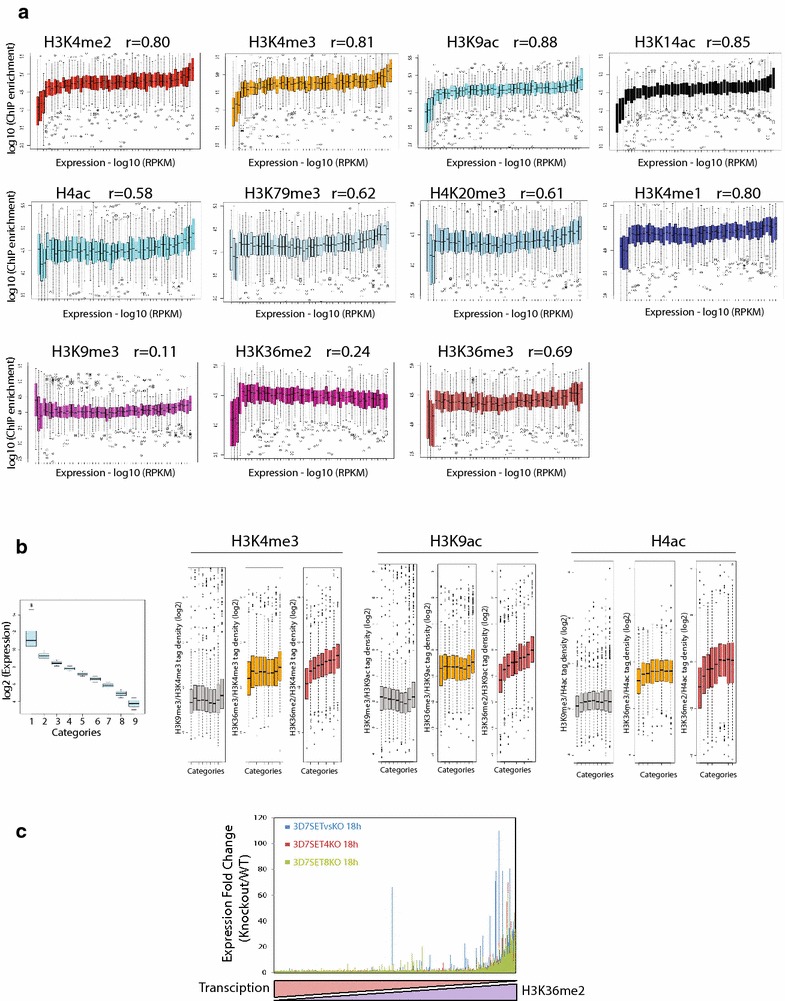
Correlation of histone modifications and transcription in *P. falciparum.* **a** Genes were categorized based on increasing expression levels (based on reads assigned per kilobase of target per million mapped reads (RPKM); enrichments were collected at corresponding genes and Whisker plots were plotted for each category. Whisker plots representing the enrichment of histone modifications (H3K4me2, H3K4me3, H3K9ac, H3K14ac, H4ac, H3K79me3, H4K20me3, H3K4me1, H3K9me3, H3K36me2 and H3K36me3) at the trophozoite stage. Pearson correlation (r) was calculated between the median of gene expression and histone enrichment. Gene expression positively correlates with all histone modifications. Least correlation with transcription was observed with H3K9me3 and H3K36me3 as compared to other histone modifications. **b** Genes were categorized based on decreasing expression levels (based on RPKM), enrichments were collected at corresponding genes and occupancies of multiple potential repressive histone modifications (H3K9me3, H3K36me2 and H3K36me3) to that of activation marks (H3K4me3, H3K9ac and H4ac) were plotted for each category at trophozoite stage. Linear increase in the ratio of activation to repression mark observed with H3K36me2 suggesting that it is a global repressive mark in *P. falciparum*. **c** Fold change in expression was calculated in SET4KO, SET8KO and SETvsKO as compared to wild-type conditions and plotted as a function of decreasing transcription and increasing H3K36me2

Multiple recent studies on histone modifications led to systematic understanding of transcription regulation in various organisms, resulting in the histone code hypothesis [23]. To understand the correlation between histone modifications and transcription, we asked if the occupancies of various histone modifications correlate with gene activity in *P. falciparum*. We systematically calculated the enrichment levels of histone modifications in the gene body of all the *P. falciparum* genes at trophozoite stage and compared them with relative gene expression categorized based on RNA sequencing (Fig. 3a). As expected, strong positive correlation with transcription was observed with the active histone modifications H3K4me3 (Pearson correlation 0.81), H3K4me2 (Pearson correlation 0.80), H3K4me1 (Pearson correlation 0.80), H3K9ac (Pearson correlation 0.88) and H3K14ac (Pearson correlation 0.85). Other histone modifications such as H3K36me3 (Pearson correlation 0.69), H3K79me3 (Pearson correlation 0.62), H4K20me3 (Pearson correlation 0.61) and H4ac (Pearson correlation 0.58) showed moderate positive correlation with transcription (Fig. 3a). Surprisingly, occupancies of H3K79me3, H4K20me3 and H4ac were higher on a subset of least expressed genes, which were also enriched with H3K9me3, suggesting a novel role of these modifications on inactive genes. Interestingly, H3K36me2 (Pearson correlation 0.24) shows least correlation as compared to other known active histone modifications suggesting that it could presumably act as a global mark of gene suppression like known repression mark H3K9me3 (Pearson correlation 0.11) in *P. falciparum* (Fig. 3a).

Out of the two known repressive marks, H3K27me3 is absent and H3K9me3 occurs only on a subset of genes in *P. falciparum* [16]. Thus, we wondered which modification might serve as a global repressive mark in *P. falciparum*. Furthermore, there is no negative correlation of any histone modification with gene expression. This prompted us to calculate the ratio of the occupancies of various potential repressive histone modifications (H3K9me3, H3K36me2 and H3K36me3) to activation marks (H3K4me3, H3K9ac and H4ac), which might play an important role towards gene regulation in *P. falciparum*. To test this hypothesis, we divided *P. falciparum* genes at trophozoite stage into nine categories ranked on the basis of their expression level and then systematically calculated the ratio of enrichment levels of putative global repressive to activation marks (Fig. 3b). Any histone modification profile that exhibits linear increase in the ratio of occupancies of repressive to activation mark along with decreasing transcription is considered to be globally repressive mark. Surprisingly, our analysis reveals that H3K36me2 serves as the global repressive mark in *P.falciparum* (Fig. 3b). To further confirm this observation we took advantage of microarray-based gene expression data available for knockout of HMTs (SETs, responsible for H3K36 methylation) in *P. falciparum* [8]. We sorted all *P. falciparum* genes based on their expression levels and calculated the fold change in gene expression upon knockout of HMTs SETvs, SET4 and SET8 (responsible for H3K36 methylation [8]) as compared to the wild-type conditions (Fig. 3c). Interestingly, knockout of HMT genes leads to higher expression of least expressed genes with highest H3K36me2 in the wild-type condition. Moreover, we compared the relative expression between wild-type and SETvs knockout conditions and observed a global up-regulation of genes (Additional file 1: Figure S5) suggesting that H3K36 methylation is indeed involved in gene silencing. Further to confirm that H3K36me2 is a global repressive mark, we calculated the correlation between transcription level and histone marks after the exclusion of virulence genes (first three subsets in Fig. 3a, which are also enriched with H3K9me3). Interestingly, H3K36me2 shows negative correlation with expression (Pearson correlation −0.79) when the virulence genes are excluded (Additional file 1: Table S4). This further strengthens our conclusion that H3K9me3 acts as a repressive mark on a subset of genes whereas H3K36me2 (in a mutually exclusive manner) acts as global repressive mark and gene expression is probably governed by a novel mechanism of the ratio of repression to activation marks in *P. falciparum.*

### Histone modification profiles indicate widespread anti-sense and divergent transcription in *P.falciparum*

**Fig. 4:**
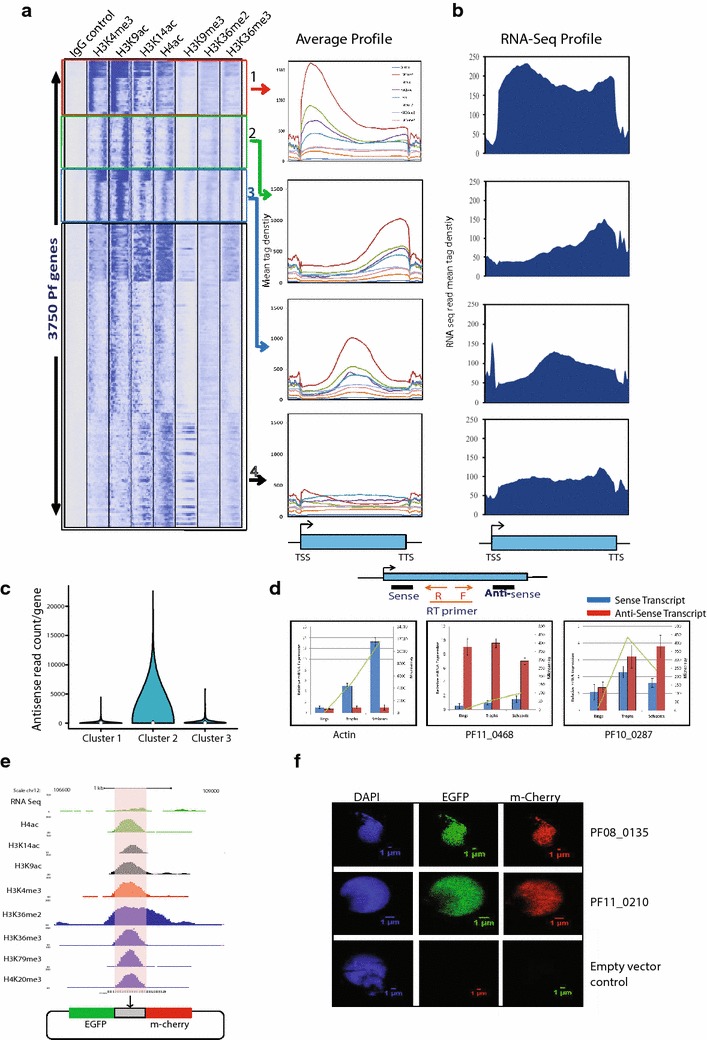
Clustering of *P. falciparum* genes based on histone modification profiles. **a** Heat map of the signal density using k-means clustering observed on 3750 *P. falciparum* genes for IgG control and histone modifications (H3K4me3, H3K9ac, H4ac, H3K9me3, H3K36me2, and H3K36me3). Genes were clustered into four distinct categories based on the distribution of histone modifications in the gene body: (1) Cluster 1 (411 genes), peaks at the 5′ end of the gene (red squared and labeled 1), Cluster 2 (562 genes), peaks at the 3′ end of the genes (*green squared* and labeled 2), Cluster 3 (335 genes), peaks observed at the center of the gene body (*blue squared* and labeled 3) and Cluster 4 (2442 genes), with very low levels of histone modifications (*black squared* and labeled 4). **b** RNA sequencing (RNA-seq) profiles of the respective cluster plotted as average gene unit. RNA-seq reads correlate with the histone modifications. **c** Levels of naturally anti-sense transcript calculated for cluster 1, 2 and 3 using publicly available data. Cluster 2 shows maximum anti-sense transcript levels as predicted with histone modification profiles. **d** Sense and anti-sense transcript were measured for PF11_0468 and PF10_0287 genes from cluster 2. Actin (from cluster 1) was used as control. Both these genes showed significant anti-sense transcript production. **e** UCSC snapshot of a representative gene having histone modifications at the center of the gene unit. The intragenic region was cloned in a dual promoter reporter vector in which mCherry and EGFP were cloned in sense and anti-sense orientation. **f** Intragenic region showed both EGFP and mCherry expression suggesting bidirectional promoter activity from the two randomly selected genes. Differential levels of mCherry and EGFP between two intragenic sequences indicate differential promoter strength of these sequences. Empty vector backbone was used a negative control

Distribution patterns of histone modifications in gene body are predictive of gene functions. To understand the differential functions of histone modifications distribution, we generated the composite profiles of input control, representative histone modifications H3K4me3, H3K9ac, H4ac, H3K9me3, H3K36me2 and H3K36me3 in the entire gene body and extending 1.5 kb upstream and 1.5 kb downstream of 3750 genes expressed at the trophozoite stage. Genes were clustered into four distinct categories based on the distribution of histone modifications in the gene body: (1) Cluster 1 (411 genes), peaks at the 5′ end of the gene (red squared and labeled 1), Cluster 2 (562 genes), peaks at the 3′ end of the genes (green squared and labeled 2), Cluster 3 (335 genes), peaks observed at the center of the gene body (blue squared and labeled 3) and Cluster 4 (2442 genes), with very low levels of histone modifications (black squared and labeled 4) (Fig. 4a, Additional file 2: Table S5). Histone modification profiles were further correlated with RNA-seq reads (Fig. 4b) and gene ontology terms (Additional file 1: Figure S6). Genes in cluster 1 have equal distribution of RNA-seq reads in the gene unit, which in turn indicates that these genes are transcribed as a single transcript. Furthermore, genes in cluster 1 are highly expressed and associated with housekeeping functions and cell growth (Additional file 1: Figure S6, S7). In cluster 2, histone modification peaks correlate with the RNA-seq reads suggesting that these genes might be involved in production of anti-sense transcripts. To correlate our data with the existing natural anti-sense transcript data [17], we monitored the levels of anti-sense transcripts produced from the first three clusters. In agreement with our prediction, maximum anti-sense transcripts were observed in cluster 2 as opposed to cluster 1 and 3 (Fig. 4c). To further confirm this, we randomly selected genes from cluster 2 and monitored sense and anti-sense transcription. We found that genes with peaks of histone modifications at the 3′ end produce sense and anti-sense transcripts (Fig. 4d). Together, it suggests that genes in cluster 2, which are mostly associated with evasion or tolerance of host immune response (Additional file 1: Figure S6), are regulated by anti-sense transcripts. In cluster 3, again we found RNA-seq reads correlating with the histone modification peaks in the center of gene body indicating presence of alternative or bidirectional promoters (Fig. 4a, b). To verify the promoter activity of these genomic regions, we designed a dual reporter vector in which mCherry and EGFP were cloned in sense and anti-sense orientation to genomic elements with predicted promoter activity (Fig. 4e). Interestingly, when these constructs were transfected into *P. falciparum,* the expression of both mCherry and EGFP was observed suggesting that these genomic regions within the gene body possess indigenous bidirectional promoter activity (Fig. 4f). Furthermore, the levels of mCherry and EGFP are comparable for the given bidirectional promoter (intragenic sequence) but not comparable to different sequences indicating differential promoter strength of these sequences (Fig. 4f). It is also possible that these bidirectional promoters exhibit stage specificity. Interestingly, both mCherry and EGFP were localized to the nucleus which might be result of their translocation because of small size as suggested earlier [24]. On the other hand, transfection of same constructs in HeLa human cervical epithelial cells did not reveal any expression highlighting differences in the recognition of transcription start site between human and *P. falciparum* (data not shown). Genes in cluster 3 are associated with stimulus-dependent functions (Additional file 1: Figure S6) indicating their regulation by bidirectional promoters. Finally, cluster 4, which represents maximum numbers of genes, has low levels of histone modifications. Genes in cluster 4 are least expressed and associated with functions such as entry into host (Fig. 4a, b, Additional file 1: Figure S6). Thus, our analysis suggests that *P. falciparum* has expanded its repertoire of gene regulation by anti-sense RNA and bidirectional promoters. Our analysis also demonstrates that histone modification profiles can be used to predict the differential function of genes, and this epigenomic landscape can be further used to overcome many conceptual gaps in our understanding of *P. falciparum* transcription.

### Clonally variant multicopy genes harbor distinct epigenetic signatures

**Fig. 5:**
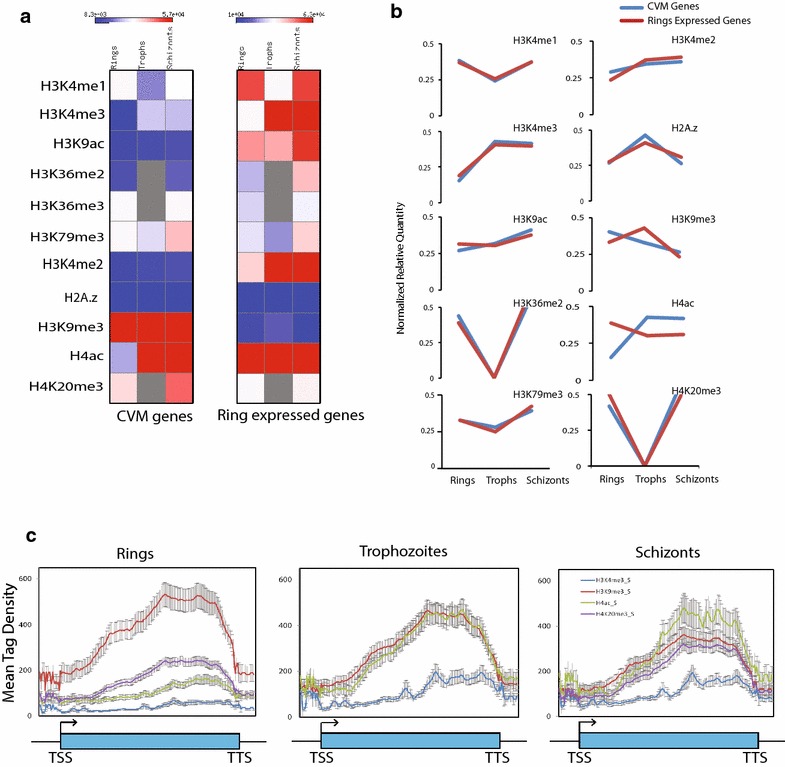
Histone modification profiles over CVM genes. **a** Enrichment of various histone modifications and histone variant H2A.z on CVM and 500 ring-expressed genes calculated in a stage-specific manner. **b** Stage-specific profile of histone modifications across different IEC stages in CVM and ring-expressed genes. H3K9me3 and H4ac showed differential pattern over CVM and ring stage-expressed genes. **c** Average profiles of H3K9me3, H4ac and H4K20me3 histone modifications over CVM genes, which were found to be differentially distributed between CVM and ring-expressed genes. *Error bars* (*black*) standard error of the mean

*Plasmodium falciparum* contains several clonally variant multicopy (CVM) gene families such as *var*, rifin and stevor, which are shown to play a central role in enabling host immune evasion and promoting pathogenesis. These multicopy gene families exhibit monoallelic expression, which is shown to be regulated by epigenetic modifications [25, 26]. However, mechanism of selection of a single gene for expression including the involvement of transcription factors and genetic elements is still not clear. As histone modifications are predictive of gene functions, we decided to examine the epigenetic signatures at *var* and rifin genes. We calculated the enrichment of multiple histone modifications (H3K4me1, H3K4me2, H3K4me3, H3K9ac, H3K36me2, H3K36me3, H3K79me3, H3K9me3, H4ac and H4K20me3) and histone variant H2A.z on CVM genes and compared them with 500 ring-expressed genes in stage-specific manner (Fig. 5a). Our analysis suggests that occupancies of most of the histone modifications (H3K4me1, H3K4me2, H3K4me3, H3K9ac, H3K36me2, H3K36me3 and H3K79me3) and histone variant H2A.z are lower on CVM as compared to ring-expressed genes at different IEC stages. As the expression of CVM genes is lower as compared to other ring-expressed gene, we plotted profiles of histone modifications and histone variant H2A.z across different IEC stages. Interestingly, all histone modifications and histone variant H2A.z follow similar pattern of occupancy across different IEC stages in CVM and ring-expressed genes except H3K9me3 and H4ac histone modifications (Fig. 5a, b). We further monitored the distribution of H3K9me3 and H4ac histone modifications and H3K4me3 on CVM gene body in stage-specific manner (Fig. 5c). Interestingly, H3K9me3 and H4ac, two prominent histone modifications on the CVM genes are present at the center or 3′ end of the gene unit, with H4ac variable between different stages (Fig. 5c). H4K20me3 is found to be significantly enriched on CVM genes, however, levels of H3K4me3 and H3K9ac (activation marks) and H3K36me2 (global repressive mark) are significantly lower on CVM genes (Additional file 1: Figure S8). This in turn suggests that CVM genes are poised and regulated by different set of activation and repression marks as evidenced by enrichment of H4ac and H3K9me3, respectively. Thus, our analyses suggest that CVM genes are enriched with unique set of epigenetic marks indicating a differential combinatorial mode of transcription regulation.

## Discussion

Recent advances in genome-wide studies have opened opportunities to understand the epigenetic and transcriptional regulation in greater details. However, most of the genome-wide studies in *P. falciparum* were performed using ChIP-on-ChIP [18, 20, 27], which has several limitations including low-depth of sequencing and variable signal intensity. To perform highly quantitative and qualitative epigenome analysis at high resolution and in truly genome-wide manner, we chose next-generation sequencing platform. In particular, we used a ligation-based method suitable for sequencing homopolymeric stretches as seen in *P. falciparum* for next-generation sequencing library amplification [28], enabling highly quantitative analysis of the extremely AT-rich *P. falciparum* genome.

### *Plasmodium falciparum* exhibits distinct epigenomic landscape in comparison with its host

In this study, by contrasting the histone modifications from *P. falciparum* and its human host, we studied the differences in mechanism(s) of transcription regulation between them. Our comprehensive histone modification analysis shows that most of the chromatin in *P. falciparum* is poised, indicative of the fact that parasites undergo closed mitosis and their chromosomes do not condense during nuclear division [29]. In human cells, H3K4me3 is linked to transcription initiation, and it works in coordinated manner with other histone modifications [30]. A mechanistic cross-talk has been proposed between H3K4me3 and H3 acetylation in several organisms. Various HAT complexes (such as p300/CBP, NuA3/4 and GCN5/PCAF) are shown to recognize H3K4me3 on chromatin by their tudor-domain and acetylate chromatin [31]. This cross-talk between H3K4me3 and H3 acetylation is supported by knockdown of WDR5 (subunit of several chromatin modifying complexes including HMTs and HATs) or Set1, (catalytic subunit of H3K4 methyltransferases complex), which not only decreased H3K4me2/3, but also decreased the acetylation levels at given promoters [32]. Thus, H3K4me3 seems to provide a binding platform for HATs, which are specific for the H3 tails, but might not be specific for any particular lysine residue [30]. In concordance with this observation, our analysis shows that activation marks H3K9ac and H3K14ac have similar profiles as that of H3K4me3 at the 5′end of the genes. However, occupancy of activation marks at the promoter and body of the genes is contradictory to earlier observations in *P. falciparum* [16]. We wondered if the differences in the observed histone modification profiles are due to uneven distribution of nucleosomes in *P. falciparum* as reported earlier [16, 18]. To confirm the distribution profiles of histone modifications, we calculated the ratio of histone modifications (H3K4me3 and H3K9ac) to pan-H3. Distribution profiles of these two histone modifications with and without pan-H3 normalization are comparable (Additional file 1: Figure S9), which further supports our observation that active histone modifications (H3K4me3 and H3K9ac) are associated with promoter and coding regions in *P. falciparum*. Thus, the observed discrepancy in distribution of histone modifications between this study and earlier study [16] may have arisen from the fact that for the first time we are visualizing the profiles of histone modifications on the promoters rather than on translation start site (ATG, start codon) in *P. falciparum*. Thus, our analysis suggests that all these activation marks work together to establish more open chromatin required for the regulation of transcription. However, the elongation marks H3K36me3 and H3K79me3, which are associated with active transcription and resetting of the initial chromatin in human cells [33], show moderate positive correlation with transcription in *P. falciparum* suggesting a new mode of communication between chromatin and transcription [34]. Surprisingly, maximum enrichment for the histone H2A variant H2A.z, which is often associated with active promoters in human cells, occurs on either side of the gene unit in *Plasmodium* suggesting its function in boundary maintenance. Our analyses thus corroborate other studies which suggest that *P. falciparum* has expanded its repertoire of gene regulation by anti-sense RNA [35–37] and bidirectional promoters [38] but we illustrate it for the first time at genome-wide level and demonstrate its correlation with histone modifications. Even though the function of poised chromatin remains to be established, identification of novel exonuclease-mediated degradation pathway [37] indicates that there may be RNA decay mechanisms in action in *P. falciparum*. Thus, these findings can be used to explore the differences in transcriptional regulation between the *P. falciparum* and its host to develop inhibitors against the malaria parasite.

### H3K36me2, a newly identified potential global mark of transcriptional silencing in *P. falciparum*

**Fig. 6:**
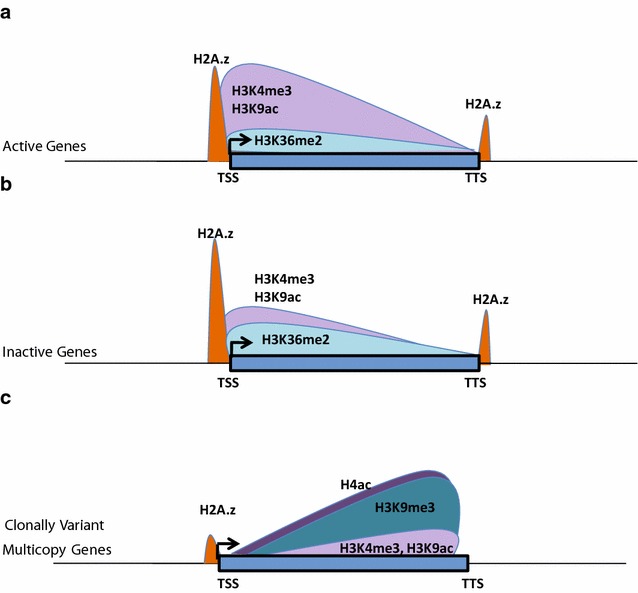
Model depicting differential occupancies of histone modifications regulating transcription of *P. falciparum* genes. Active (**a**) and inactive (**b**) genes exhibit similar profiles of histone modifications with H3K4me3 and H3K9ac as dominant marks across the gene body. On inactive genes slight increase in the levels of H3K36me2 and decrease in the levels of H3K4me3 and H3K9ac leads to alteration in the ratio of activation to repression mark, which affects gene expression. **c** Clonally variant multicopy (CVM) genes have different chromatin modifications. On CVM genes, most prominent activation and repression marks are H4ac and H3K9me3, respectively

One of the most important regulations of gene expression is via gene silencing. Gene silencing is achieved by DNA methylation or suppressive histone modifications (H3K9me3 and H3K27me3) or the interplay between these. *P. falciparum* lacks H3K27me3 histone modification (this study and [19]). Hence, H3K9me3 is the only known silencing histone mark in *P. falciparum*. However, H3K9me3 occurs only on a subset of genes, which are mostly associated with virulence and pathogenicity [16]. This in turn suggests that other layers of transcriptional repression might be involved in the silencing of *P. falciparum* genes. Our extensive genome-wide comparative analysis of histone modifications suggests that H3K36me2 is a novel global repressive mark in *P. falciparum*. Although widely perceived as an activating modification, H3K36 methylation has been shown to play a role in transcriptional repression in many organisms [39, 40]. Furthermore, our analysis shows that the ratio between activation and repression marks plays an instrumental role in regulation of gene expression in *P. falciparum*. Thus, overall profiles of histone activation and repression marks remain same on active and inactive genes but their levels vary (Fig. 6a, b). Similar mechanism of gene regulation is suggested for enhancers in higher eukaryotic system, where the strength of enhancer depends on ratio of H3K4me3 to H3K4me1 histone modifications [41]. Interestingly, H3K36me2 shows negative correlation with expression (Pearson correlation −0.79) when the virulence genes are excluded. This further strengthens our conclusion that H3K9me3 acts as a repressive mark on a subset of genes whereas H3K36me2 (in a mutually exclusive manner) acts as a global repressive mark. Future studies will be needed to define the role of H3K36me2 in gene repression and differentiate its function from H3K36me3 in *P. falciparum.* Thus, our study not only identified a global gene repressor mark in *P. falciparum*, it has also revealed a unique mode of regulation of gene expression by altering the ratio of epigenetic marks.

### Distinct regulation of clonally variant multicopy genes with unique epigenetic signatures

Most of the knowledge of clonally variant multicopy (CVM) genes has been acquired through the study of *var* genes. Many mechanisms have been suggested for the regulation of *var* genes, including involvement of non-coding RNAs [36], bidirectional promoters [38], intronic genetic elements [42], insulator elements [43], transcription factors [20] and unique epigenetic signatures such as H3K9me3 [44], H3K36me3 [8] and H2A.z exchange [45]. In this study, by doing a comprehensive analysis, we found that CVM genes are poised and have differential chromatin modifications than housekeeping genes (Fig. 6). Further, most of the histone modifications occur either in the center or at the 3′ end of the virulence genes, indicative of their regulation by a bidirectional promoter or an anti-sense RNA, respectively (Fig. 6c). This observation is in accordance with earlier study which suggests that non-coding RNAs are produced from both active and silent *var* genes [46]. Interestingly, H3K36me2, the global repressive mark identified in our analysis occurred minimally on CVM genes indicating a mutually exclusive role for the two repression marks H3K36me2 and H3K9me3. Interestingly, involvement of histone modifications (H3K4 methylation and hyperacetylation of H3 and H4) has been shown to play role in boundary maintenance at insulator elements [47]. Thus, our findings have important implications on the processes that promote immune escape and pathogenesis in human malaria parasite. Our future research will focus on the molecular machinery that can read these marks and translate this information into coordinated gene expression pattern of CVM genes.

## Conclusions

In this study, we have generated high-resolution genome-wide profiles of several histone modifications and integrated them with publicly available data for histone modifications in *P. falciparum*. The comprehensive epigenomic map generated in this study is in concordance with earlier expression analyses suggests that these data can be used to overcome many conceptual gaps in our understanding of *P. falciparum* transcription. We also found that transcriptional regulation by the virtue of poised chromatin and differential histone modifications is unique to *P. falciparum*, which could have evolved for higher rate of transcription. Furthermore, for the first instance, we identified H3K36me2 as a global repressive mark in *P. falciparum* and revealed a unique mode of regulation of gene expression by altering the ratio of activation and repression marks. Importantly, the observed differences in the epigenetic code and transcriptional regulation in *P. falciparum* and its host will open new avenues for targeting the epigenetic machinery for drug development in malaria as well as other related parasites in future.

## Methods

### Parasite culture

*P. falciparum* strain 3D7 was cultured as previously described [48]. Briefly, parasites were cultured in RPMI1640 medium supplemented with 25 mM HEPES, 0.5 % AlbuMAX I, 1.77 mM sodium bicarbonate, 100 μM hypoxanthine and 12.5 μg ml^−1^ gentamicin sulfate at 37 °C. RBCs were prepared from fresh whole blood obtained from a healthy donor and stored at 4 °C for at least 1 day. Increased yields were obtained by subculturing the cells every 2 days for 6–8 h before invasion. This was achieved by equally dividing the contents of each flask into two or more flasks and quickly restoring the hematocrit between 1 and 1.5 % in the required volume of culture medium. The culture was synchronized with 5 % sorbitol and the parasites were collected at 18, 30 and 40 hpi for chromatin immunoprecipitation.

### Antibodies

H3K4me2 (Upstate 07-030), H3K4me3 (Upstate 07-443), H3K9ac (Upstate 06-942), H3K9me3 (Upstate 07-442) antibodies were validated earlier in *P. falciparum* [16, 49]. H3K4me1 (Upstate 07-436), H3K14ac (Upstate 07-353), H3K27ac (Abcam Ab4729), Pan-H4ac (Upstate 06-866) and H3K79me3 (Upstate 07-952) antibodies were found positive in ChIP-qPCR test (data not shown) and were further tested for their specificity in Western blot on *P. falciparum* lysate (Additional file 1: Figure S10). For IgG (Millipore 12-370) and H3K27me3 (Upstate 07-449) antibodies were used for ChIP-sequencing.

### Chromatin immunoprecipitation (ChIP) and sequential ChIP

Infected RBCs were crosslinked with 1 % formaldehyde (Catalog number—28908, THERMO Scientific) for 10 min, lysed and sonicated in sonication buffer (10 mM Tris–HCl pH 7.5, 200 mM NaCl, 1 % SDS, 4 % NP-40, 1 mM PMSF) to obtain an average chromatin size of 200–400 bp. Chromatin was pre-cleared using 50 µl of a 50 % protein A Sepharose (GE healthcare) slurry for 1 h at 4 °C with gentle inverting. Immunoprecipitations were carried out in 1 ml of IP buffer (20 mM Tris–HCl pH 8.0, 150 mM NaCl, 2 mM EDTA, 1 % Triton-X 100). Three µg antibody was used per 20 µg purified chromatin. Input chromatin was obtained after preclearing by de-crosslinking and purified using the Qiaquick column (Qiagen) according to manufacturer’s instructions. Immunoprecipitations were carried out with inverting at 4 °C for 14–16 h. The samples were then incubated with 50 µL of a 50 % Protein A Sepharose slurry for 3 h at 4 °C with gentle inverting. IP samples were reverse-crosslinked and the DNA was purified using a Qiaquick column (Qiagen). Target sites obtained from ChIP-seq analysis were further validated by quantitative PCR using Power SYBR Green Master Mix (Applied Biosystems). For sequential ChIP, at least four ChIP assays (4 × 20 µg purified chromatin) were used for the first IP (H3K9ac). Following standard washing, elution was performed with 10 mM DTT (30 min, 37 °C). The eluates from four ChIPs were combined, diluted at least 30 times with ChIP dilution buffer and secondary antibody (H3K4me3) was incubated overnight. The subsequent steps were performed as for regular ChIP.

### Library preparation and sequencing

ChIP-seq libraries for all the samples were prepared from around 5 ng of DNA using fragment library construction kit from Life technologies for SOLiD sequencing. Briefly ChIPed DNA samples were end repaired and adapters were ligated using T4 DNA ligase. Ligated DNAs were subsequently amplified using adapter specific bar-coded primers for 15 cycles. DNA purification at every step was performed using Agencourt XP beads (Beckman Coulter). Library profiles were assessed using Agilent bioanalyzer 2100 high-sensitivity DNA kit. Equimolar amount of libraries were pooled and 50 bp reads were sequenced in-house using SOLiD 4.0 sequencer (Applied Biosystems).

### *Plasmodium falciparum* ChIP-seq data processing

Reference genome (PlasmoDB-9.3_P.falciparum 3D7) was converted to color space using bowtie-build. ChIP-seq data were mapped using the Bowtie allowing two mismatches. Bed files were generated using Samtools [50]. ChIP-sequencing data were not normalized for the reads as the numbers of reads were comparable between different stages (Additional file 1: Table S1).

### Creation of density files for genome browser data visualization

Raw BED files are used as input for ad hoc (WIG) density file creation script as described in [51]. Reads are directionally extended of their theoretical length (200 bp), and 25 bp bins are created. In each bin, the maximal number of overlapping reads is computed. Tracks were uploaded and displayed using fixed-scale representation in the University of California Santa Cruz (UCSC) genome browser [52].

### Transcriptome assembly and transcription start site (TSS) and transcription termination site (TTS) identification in *P. falciparum*

To determine the Transcription Start Site of coding gene in *Plasmodium falciparum*, a reference-based transcriptome was assembled using previously reported RNA-seq data covering 8 samples collected at different time points of erythrocytic cycle stage [18]. Here, Pfalciparum3D7_Genome_v9.3 genome index was built by bowtie2-build (version 2.2.3) and all raw reads from RNA-seq results from different stages were aligned using TopHat (v2.0.13) to indexed genome [53]. In the alignment process a total of 89,645,433 (76.6 %) reads were mapped out of 117,030,592 reads. Coverage of aligned reads was calculated from outfile generated from mpileup option present in samtools [54] and this shows average coverage of 256.53. Assembly of the transcripts from aligned reads was carried out by cufflinks (v2.2.1) program. Cufflinks program was ran with following parameters, minimum intron length set as 10 nt and a maximum intron length as 10000 nt and assembly was guided by GFF file (PlasmoDB-9.3_Pfalciparum3D7.gff) to annotate the transcripts [53]. From the assembled transcripts (GTF file) gene start and end coordinates were extracted and used as TSS and TTS for further analysis. A list of unfiltered new TSS and TTS along with mapped Ids and old TSS and TTS were given in Additional file 3: Table S6.

### Validation of new TSS

Validation of the identified TSS from the current analysis was carried out by comparing with 5 prime EST sequence data available at http://fullmal.hgc.jp/ [55]. A list of new TSS with a minimum 100 bp and maximum of 2000 bp upstream to ATG (start codon) of reported gene model (PlasmoDB-9.3_Pfalciparum3D7.gff) was selected for this analysis. Here, 5 prime EST sequences were aligned to *Plasmodium falciparum* genome (PlasmoDB-9.3_Pfalciparum3D7_Genome.fa) using BLAT tool [56] and EST alignment showing more than 98 % identity were selected for further processing. Genomic co-ordinates of confidently aligned 5 prime EST sequences were selected and a separate BED file was made using AWK programming language. This BED file was sorted and intersected with another BED file of newly identified TSS (genomic co-ordinates of TSS and 100 bp downstream) from present study using intersection option available in BEDTools [57]. Resulting intersection file validates a minimum of 147 TSS sites based on their overlap. Representative alignments are shown in Additional file 1: Figure S11.

### Average profile calculations

We extracted the tag density in a 1.5 kb window surrounding the TSSs and gene body using the program seqMINER which generates heatmap as well as the profiles [51]. The sequenced ChIP-seq reads represent only the end of each immunoprecipitated fragments instead of the precise protein–DNA binding sites. To illustrate the entire DNA fragment, essentially before analysis, 3′ end of each ChIP-seq read was extended to 200 bp in the direction of the reads. For average gene profiles, genes (±1500 bp from binding site) were divided into 100 bins of length relative to the gene length. Moreover, 10 equally sized (50 bp) bins were created on the 5′ and 3′ of the gene and ChIP-seq densities were collected for each dataset in each bin. Data are normalized by dividing the reads per bin by total reads per modification.

### Data source and analysis

ChIP-seq datasets were downloaded from the public data bank Gene Expression Omnibus (http://www.ncbi.nlm.nih.gov/gds) under the accession number: GSE23867 (*P. falciparum*) and GSM840279 (Human); (Additional file 1: Tables S2, S3). The scatter plots, k-means clustering and average gene profiles are created using seqMiner [51]. Box plots and correlation analysis are produced using ‘R’ software (http://r-project.org/). UCSC genome browser was used for data visualization [52]. Number of mapped reads for each data set is provided in Additional file 1: Table S1. Sequences of primers used for quantitative ChIP-PCR validation are listed in Additional file 1: Table S7.

### Histone modification and expression correlation

Genes were categorized based on increasing expression levels (based on reads assigned per kilobase of target per million mapped reads (RPKM)) using the RNA-seq data (GSE23865, [18]). Datasets for different stages were normalized for the total number of uniquely mapped tags. Stage-specific RNA-Seq data were corrected for each stage by a correction factor (based on the amount of RNA per parasite nucleus present in each stage as described earlier [18]). Enrichment of each histone modification is calculated over these categories over the entire gene unit as described earlier [58]. Whisker plots were plotted using R boxplot function with default parameters for each category for all the histone modifications. Pearson correlation was calculated between the median of gene expression and histone modification for each category.

### Gene ontology analysis

Gene ontology analysis was performed using MADIBA [59]; an online web interface (http://madiba.bi.up.ac.za/). REVIGO (revigo.irb.hr) was used to summarize and visualize gene ontology terms.

### Comparison of histone modifications on CVM and ring-expressed genes

Total tag density of histone modifications was calculated as described above in average profile calculation over the CVM genes and 500 ring expressed genes in a stage-specific manner. The data were visualized using the matrix2png [60].

### Strand-specific RT-PCR

Total RNA was isolated at three different stages of *P. falciparum* growth. RNA was treated with DNaseI (Ambion) as described in the manufacturer’s protocol followed by phenol–chloroform extraction. RT-PCR was performed using 500 ng total RNA. For each gene, reverse transcription (RT) was performed using a forward and reverse primer. After first strand cDNA synthesis PCR was performed using sense and anti-sense primers. Sequences of primers used for strand-specific RT-PCR are provided in the Additional file 1: Table S7.

### Construction of pDR vector to study the bidirectional promoters

To score for bidirectional transcription of putative DNA elements from Cluster 3, we designed a dual reporter vector (pDR) using pmCherry-N1 as a backbone in which, EGFP and M-cherry were cloned in sense and anti-sense orientation to each other. Briefly, pmCherry-N1 vector was digested with Ase1 and Nhe1 to remove the CMV promoter. EGFP sequence was PCR amplified using pEGFP-N1 vector as a template, along with SV40 polyadenylation signal to make sure that there is no difference in RNA stability of Cherry and EGFP. Amplified EGFP product was purified by extraction using phenol:chloroform and subsequently digested with Ase1 and Nhe1. The restriction digestion products were purified by running on gel followed by purification by gel extraction column (Qiagen). Vector (pmCherry-N1) and insert (EGFP) were ligated and transformed into *E*. *coli* (DH5α) cells. The resulting colonies were screened via restriction digestion for presence of EGFP insert. A number of positive clones were selected and subjected for large-scale DNA purification via CsCl density gradient method. All positive clones were confirmed by sequencing. Primers used for EGFP amplification were designed such that EGFP insertion occurred in an orientation opposite to that of the DsRed monomer protein. DNA elements (from cluster 3) with predicated bidirectional activity from P. falciparum were cloned in sense and anti-sense to M-cherry and EGFP, respectively. Positive clones for each element along with the pDR vector were transfected in *P. falciparum* by electroporation. Cells were fixed and analyzed by confocal microscope to score for EGFP and M-cherry expression 48 h post electroporation. The primers used in construction of bidirectional promoters are listed in the Additional file 1: Table S8.

### Data access

All the stage-specific ChIP-sequencing data for histone modifications are submitted to Gene Expression Omnibus (GEO) database under the accession number GSE63369 (Additional file 1: Table S1).

## Additional files

**Additional file 1.** Combined Additional Figures S1–S11 and Additional Tables S1–S4, S7 and S8. **Figure S1.** Validation of H3K4me3 and H3K9ac ChIP-seq in Plasmodium falciparum trophozoite chromatin by ChIP-qPCR. **Figure S2.** Average transcribed genome coverage in ChIP-sequencing at different stages of Plasmodium falciparum growth.** Figure S3. **Comparison of H3K4me3 and H3K9ac data produced in this study with existing ChIP-sequencing data for these two histone modifications. **Figure S4.** Comparison of H3K4me3 modification profile of the data produced in this study with existing ChIP-sequencing data.** Figure S5.** Comparison of the expression between wild type and SETvs knockout conditions. **Figure S6.** Gene Ontology (GO) Analysis of the clusters based on histone modification profiles. **Figure S7.** Correlation between gene expression and length of different cultures. **Figure S8.** Clustering of P. falciparum CVM genes based on histone modification profiles. **Figure S9.** Comparison of profiles of H3K4me3 and H3K9ac after normalizing with pan-H3 over averaged Plasmodium falciparum genes. **Figure S10.** Western blot for histone modifications using Plasmodium falciparum lysate. **Figure S11.** Representative alignments of new TSSs, 5’EST with reported gene models. **Table S1.** Sequence statistics for ChIP-sequencing performed in this study of Plasmodium falciparum. **Table S2.** Sequence statistics for ChIP-sequencing and RNA sequencing from GEO data base for Plasmodium falciparum. **Table S3.** Sequence statistics and accession number of human histone modifications. **Table S4.** Correlation of histone modifications and transcription including and excluding virulence genes. **Table S7.** Primers used for ChIP-qPCR and PCR. **Table S8.** Co-ordinates and primers used for the cloning of bidirectional promoters.
**Additional file 2: Table S5.** List of genes and their co-ordinates for each cluster is provided in a separate excel file.
**Additional file 3: Table S6.** Co-ordinates for transcription start sites (TSSs) and transcription termination sites (TTSs) for all the *Plasmodium falciparum* genes.

## Abbreviations

RBCs: red blood cells
IEC: intra-erythrocytic cycle
HATs: histone acetyl transferases
HDACs: histone deacetylases
HMTs: histone methyltransferases
HDMs: histone demethylases
TSS: transcription start site
TTS: transcription termination site
