# Supplementary material for: A comprehensive epigenome map of Plasmodium falciparum reveals unique mechanisms of transcriptional regulation and identifies H3K36me2 as a global mark of gene suppression
Source: Epigenetics Chromatin. 2015 Sep 17;8:32. doi: 10.1186/s13072-015-0029-1 (PMC4574195; doi:10.1186/s13072-015-0029-1)
Supplement: Supplementary file 1 — Additional file 1. Combined Additional Figures S1–S11 and Additional Tables S1–S4, S7 and S8. Figure S1. Validation of H3K4me3 and H3K9ac ChIP-seq in Plasmodium falciparum trophozoite chromatin by ChIP-qPCR. Figure S2. Average transcribed genome coverage in ChIP-sequencing at different stages of Plasmodium falciparum growth. Figure S3. Comparison of H3K4me3 and H3K9ac data produced in this study with existing ChIP-sequencing data for these two histone modifications. Figure S4. Comparison of H3K4me3 modification profile of the data produced in this study with existing ChIP-sequencing data. Figure S5. Comparison of the expression between wild type and SETvs knockout conditions. Figure S6. Gene Ontology (GO) Analysis of the clusters based on histone modification profiles. Figure S7. Correlation between gene expression and length of different cultures. Figure S8. Clustering of P. falciparum CVM genes based on histone modification profiles. Figure S9. Comparison of profiles of H3K4me3 and H3K9ac after normalizing with pan-H3 over averaged Plasmodium falciparum genes. Figure S10. Western blot for histone modifications using Plasmodium falciparum lysate. Figure S11. Representative alignments of new TSSs, 5’EST with reported gene models. Table S1. Sequence statistics for ChIP-sequencing performed in this study of Plasmodium falciparum. Table S2. Sequence statistics for ChIP-sequencing and RNA sequencing from GEO data base for Plasmodium falciparum. Table S3. Sequence statistics and accession number of human histone modifications. Table S4. Correlation of histone modifications and transcription including and excluding virulence genes. Table S7. Primers used for ChIP-qPCR and PCR. Table S8. Co-ordinates and primers used for the cloning of bidirectional promoters. [file 13072_2015_29_MOESM1_ESM.pdf]

A comprehensive epigenome map of *Plasmodium falciparum* reveals unique mechanisms of transcriptional regulation and identifies H3K36me2 as a global mark of gene suppression

Krishanpal Karmodiya[1*], Saurabh J. Pradhan[1¶], Bhagyashree Joshi[1¶], Rahul Jangid[1], Puli Chandramouli Reddy[1] and Sanjeev Galande[1, 2]

[1]Department of Biology, Indian Institute of Science Education and Research, Pune, Maharashtra, India.

[2]National Centre for Cell Science, Pune, India

*Corresponding author

E-mail: krish@iiserpune.ac.in

[¶]These authors contributed equally to this work

Krishanpal Karmodiya: krish@iiserpune.ac.in

Saurabh J. Pradhan: saurabh.j.pradhan@gmail.com

Bhagyashree Joshi: bsjoshi.305@gmail.com

Rahul Jangid: rahulkumarjangid@gmail.com

Puli Chandramouli Reddy: pulichandramoulireddy@gmail.com

Sanjeev Galande: sanjeev@iiserpune.ac.in

Additional Figure S1. Validation of (A) H3K4me3 and H3K9ac ChIP-seq in *Plasmodium falciparum* trophozoite chromatin by ChIP-qPCR. Peaks of local enrichment of H3K4me3 and H3K9ac were determined by ChIP-qPCR. Input DNA was used to make the standard curve to determine the relative concentration of control and the promoter genomic regions (shown on the right of the graph). Fold enrichment is plotted over an arbitrarily chosen control genomic region (labeled as control). **(B)** Sequential ChIP–qPCR quantification for co-occupancy of H3K9ac (primary ChIP) and H3K4me3 (secondary ChIP) at randomly selected H3K9ac and H3K4me3 loci suggest that these loci are co-marked with H3K9ac as well H3K4me3 modifications. Enrichment after first ChIP using H3K9ac followed by re-ChIP with no antibody was used as a control. Error bars represent the standard deviation for three technical replicates. List of primers is provided in Additional Table S5.

A)

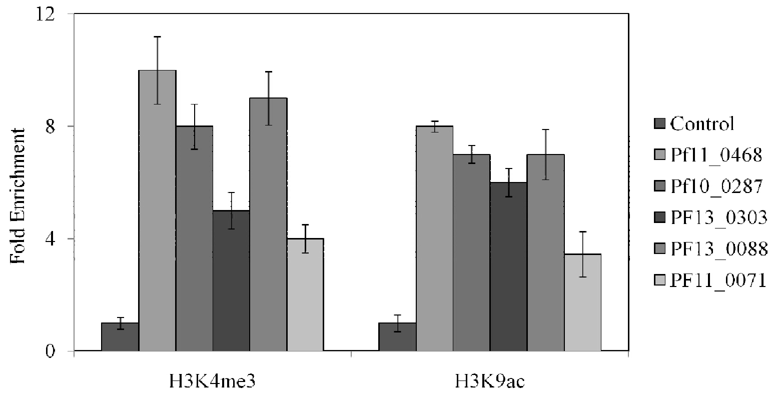

B)

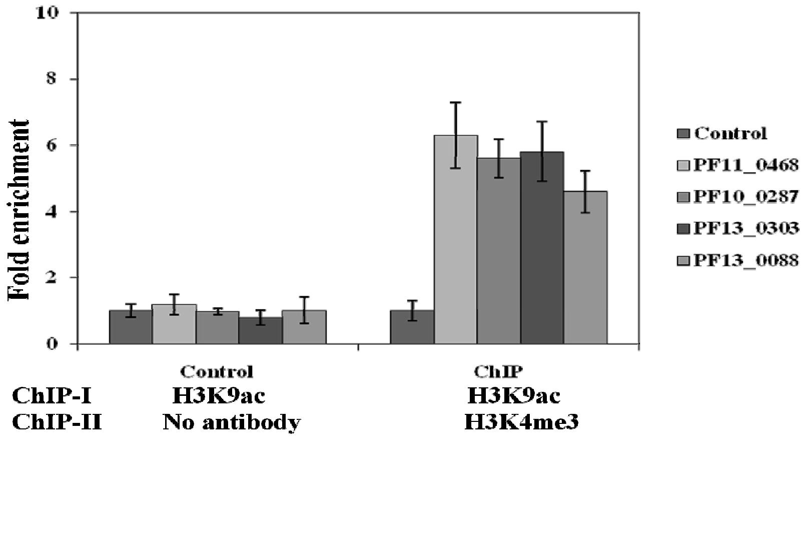

Additional Figure S2. Average transcribed genome coverage in ChIP-sequencing at different stages of *Plasmodium falciparum* growth. Transcribed genome coverage was calculated for all three stages and each histone modification. The average transcribed genome coverage obtained is ~50x.

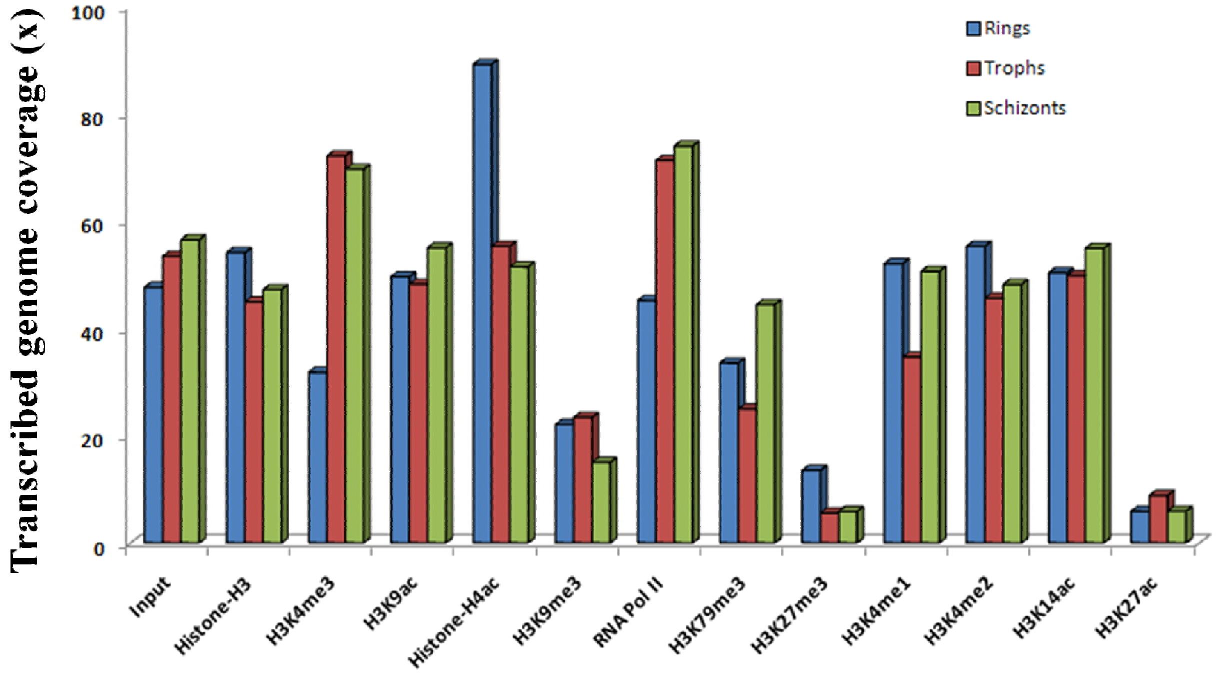

Additional Figure S3. Comparison of H3K4me3 and H3K9ac data produced in this study with existing ChIP-sequencing data for these two histone modifications. Dot plot representing enrichment of H3K4me3 and H3K9me3 data generated in this study and other study over 5,265 *Plasmodium* genes. Our data shows Pearson correlation coefficient 0.91 and 0.88 for H3K4me3 and H3K9me3 respectively with publicly available data sets (Salcedo-Amaya et al. 2009).

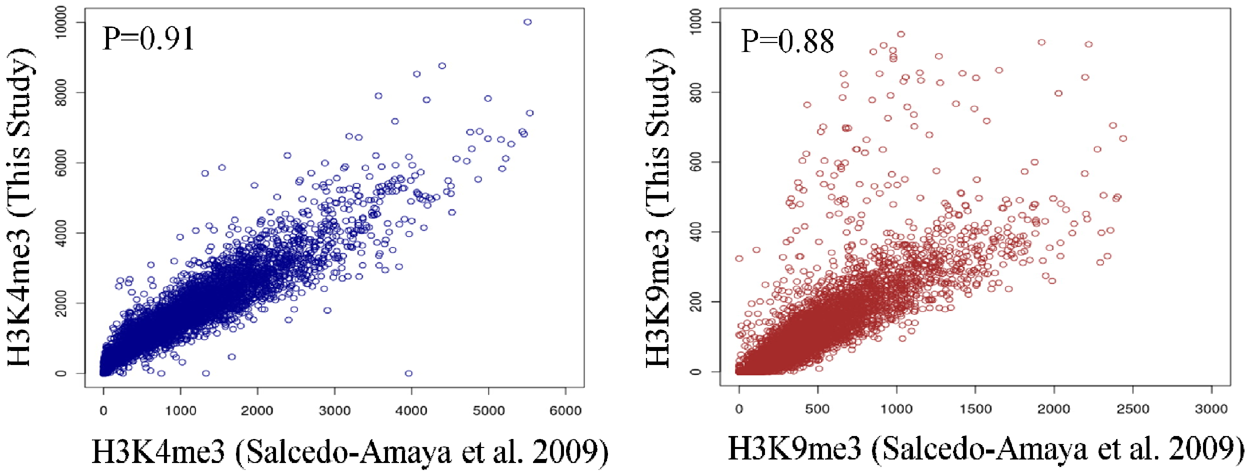

Additional Figure S4. Comparison of H3K4me3 modification profile of the data produced in this study with existing ChIP-sequencing data (Salcedo-Amaya et al. 2009). Normalized mean tag density gene profile of H3K4me3 histone modification over 5,265 *P. falciparum*. Gene unit including transcription start site (TSS) and transcription termination site (TTS) are shown below the plot. H3K4me3 has identical profile with the data produced in this study and with publicly available data (Salcedo-Amaya et al. 2009).

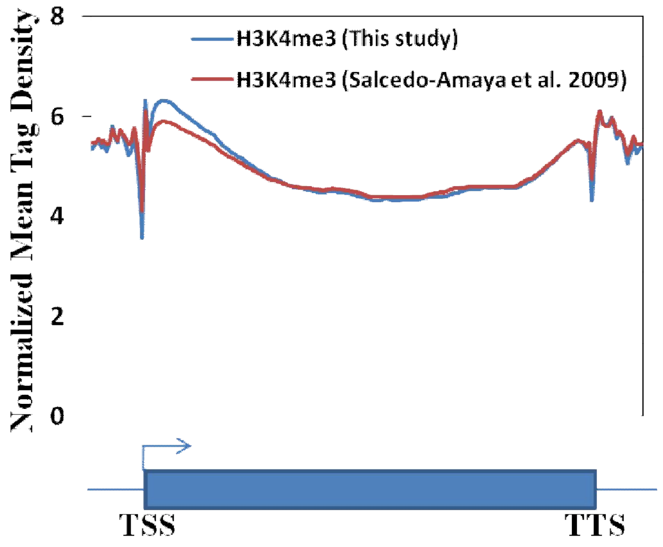

Additional Figure S5: Comparison of the expression between wild type and SETvs knockout conditions. Genes are divided into three groups based on expression levels (highest, medium and least expressed) and levels of expression were compared between wild type and 3D7SetvsKO conditions. Up-regulation of genes is observed across the categories.

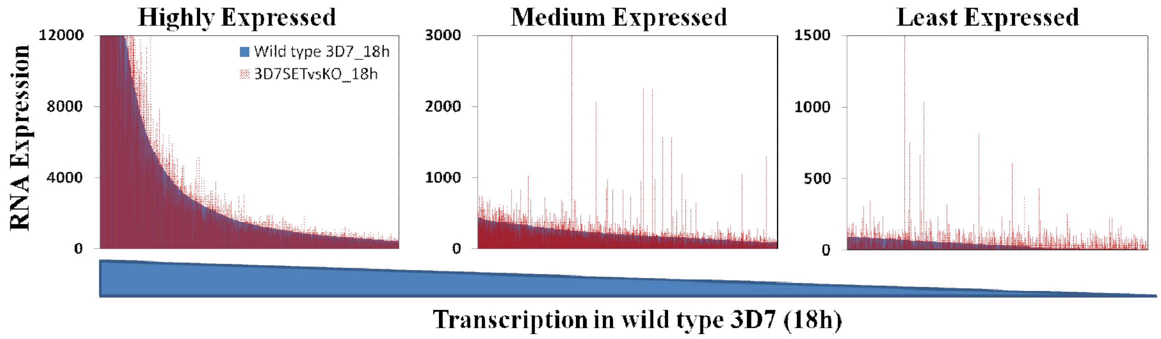

Additional Figure S6. Gene Ontology (GO) Analysis of the clusters based on histone modification profiles. Clusters described in Figure 4 are subjected to gene ontology analysis using MADIBA (Law et al. 2008); an online web interface (http://madiba.bi.up.ac.za/). REVIGO (revigo.irb.hr) was used to summarize and visualize gene ontology terms. Genes in cluster 1 are mostly associated with housekeeping functions and cell growth. Genes in cluster 2 are associated with evasion or tolerance of host immune response. Genes in cluster 3 are associated with stimulus-dependent functions. Genes in the cluster 4 associated with functions such as entry into host.

Cluster 1

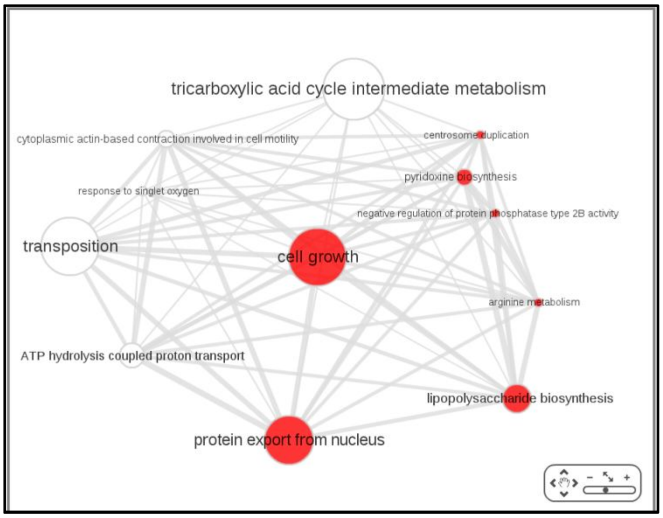

Cluster 2

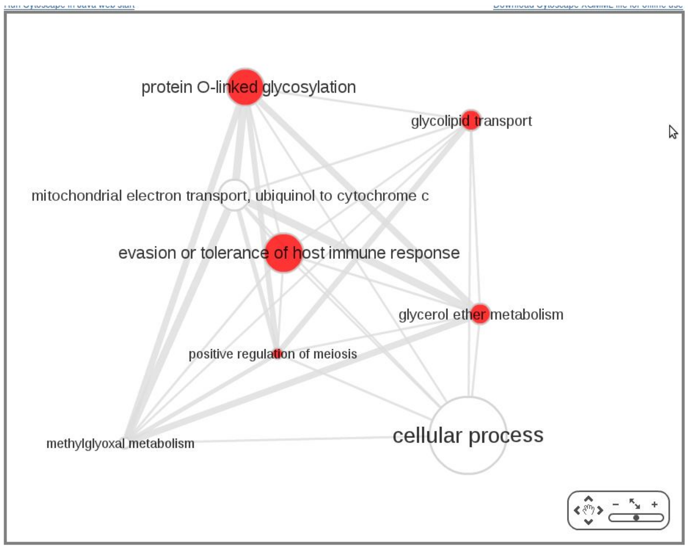

Cluster 3

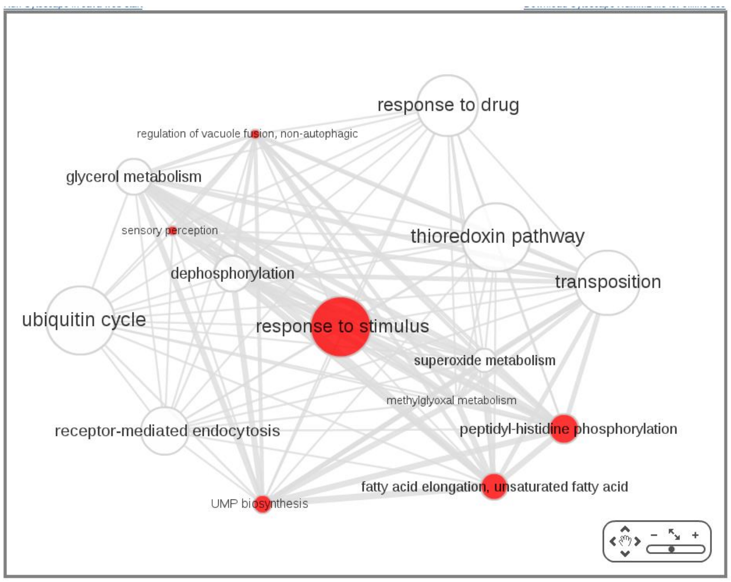

Cluster 4

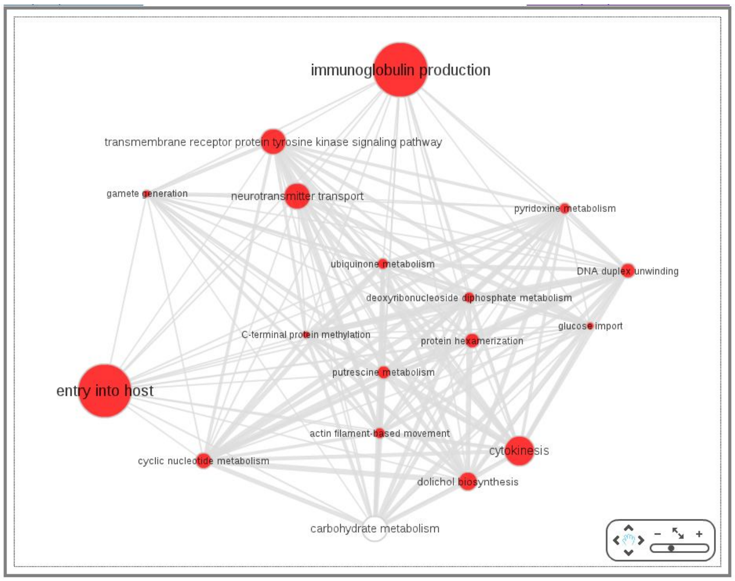

Additional Figure S7. Correlation between gene expression and length of different cultures. Box plots shows decrease in the expression levels of clusters from 1 to 4 and its inverse correlation with gene length. Further to cross-examine the correlation between gene expression and ratio of inactivation to activation mark, we calculated the ratio of inactivation and activation marks on the clusters 1 to 4.

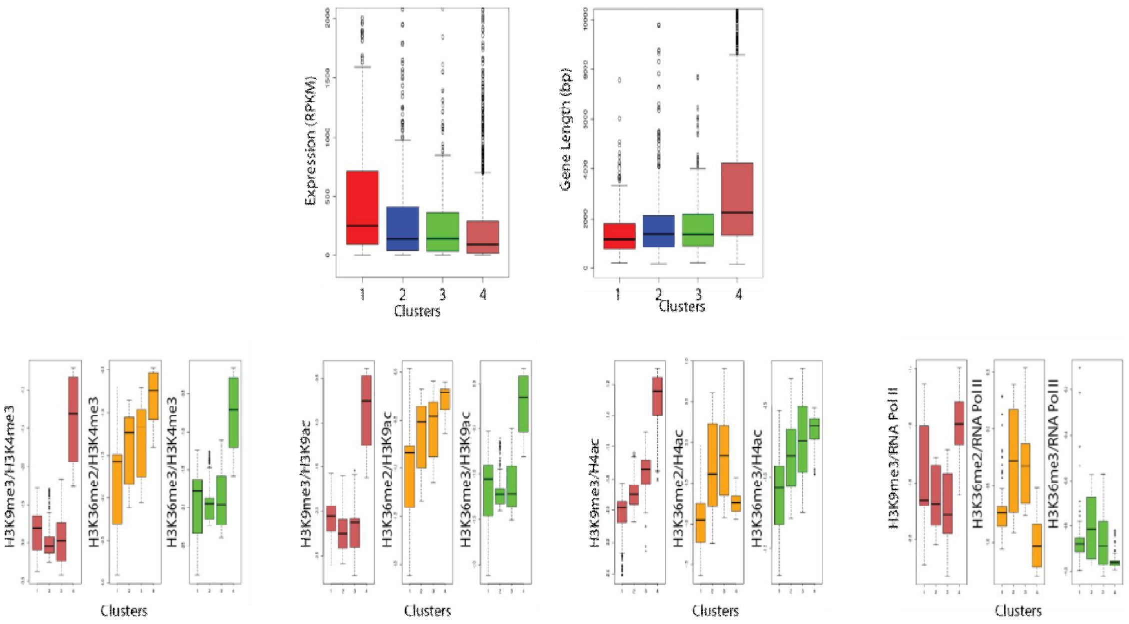

Additional Figure S8. Clustering of *P. falciparum* CVM genes based on histone modification profiles. Heatmap of the signal density using k-means clustering observed on CVM genes for histone modifications; H3K4me3, H3K9ac, H4ac, H3K9me3, H3K36me2, H3K36me3 and H4K20me3 at trophozoite stage. Average profile of genes shows on right hand site for all the CVM genes. H3K36me2 is absent on CVM genes.

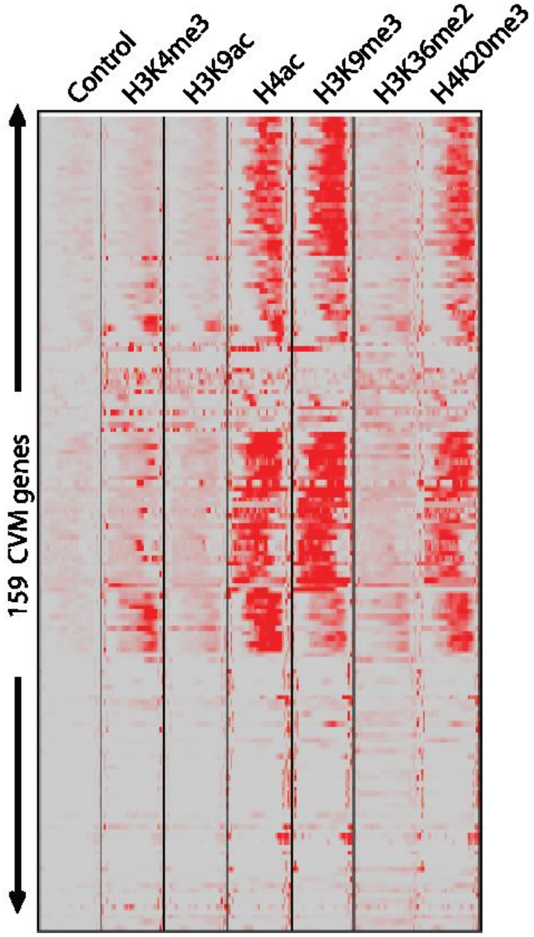

Additional Figure S9: Comparison of profiles of H3K4me3 and H3K9ac after normalizing with pan-H3 over averaged *Plasmodium falciparum* genes. (A) Mean tag density gene profile of pan-H3, H3K9ac and H3K4me3 histone modifications over 5,265 *P. falciparum* genes. (B) Profiles of H3K4me3 and H3K9ac normalized with pan-H3. Gene unit including transcription start site (TSS) and transcription termination site (TTS) is shown below. H3K4me3 and H3K9ac show comparable profiles after normalizing with pan-H3 suggesting presence of active histone modifications over the promoter and coding regions.

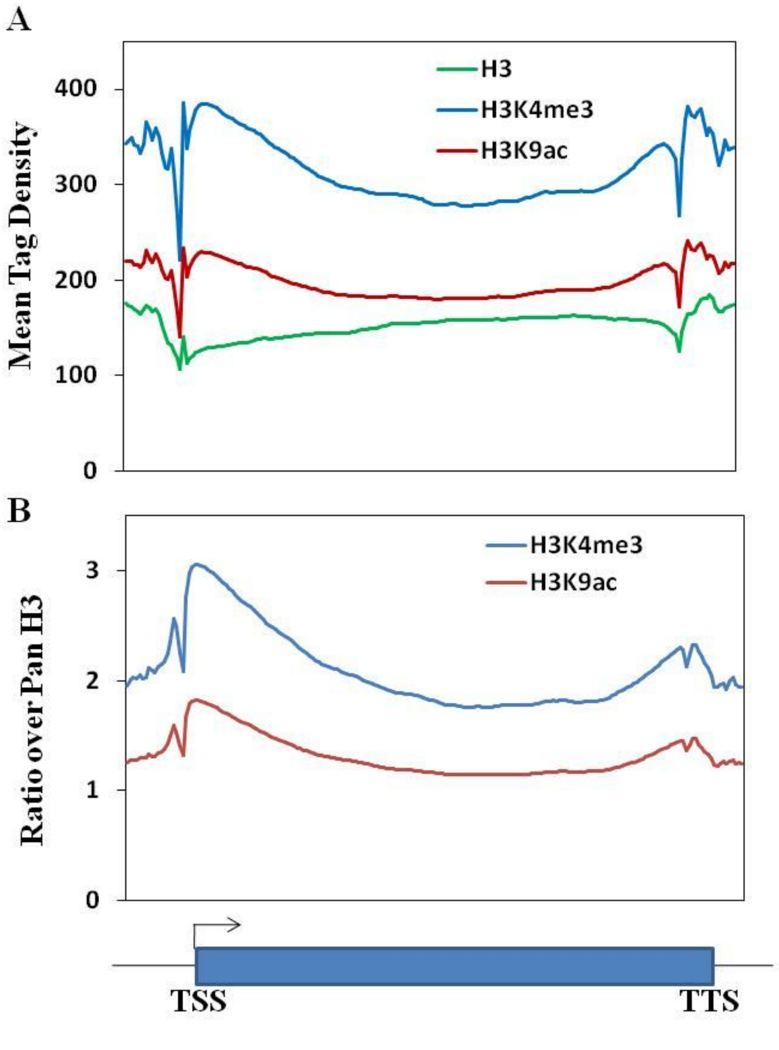

Additional Figure S10: Western blot for histone modifications using *Plasmodium falciparum* lysate.

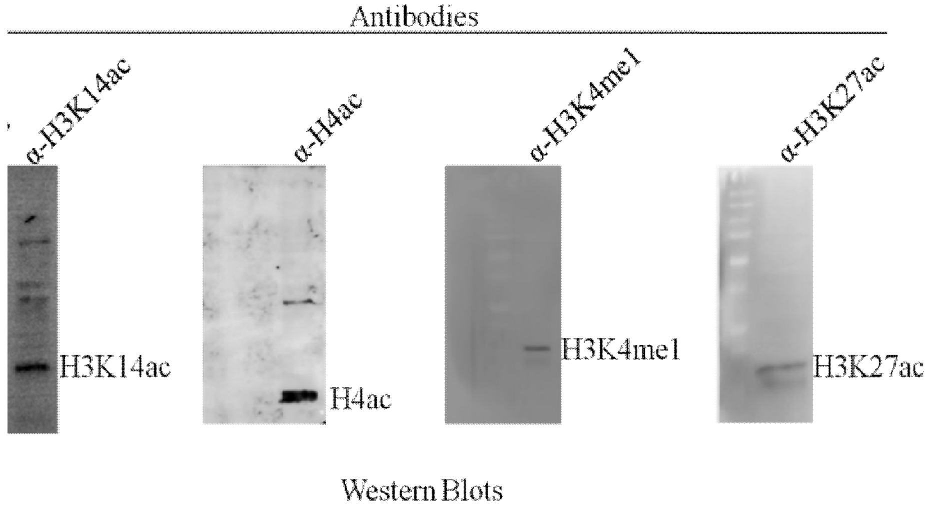

Additional Figure S11: Representative alignments of new TSSs, 5'EST with reported gene models.

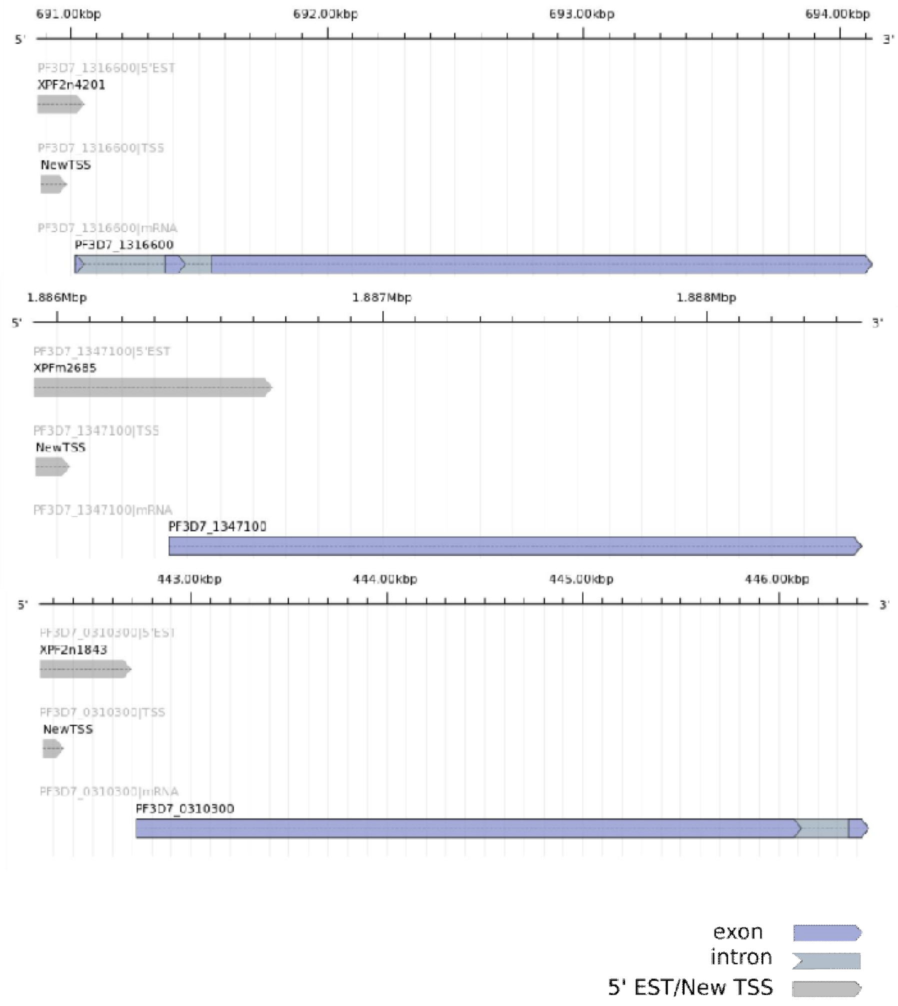

References

1. Law PJ, Claudel-Renard C, Joubert F, Louw AI, Berger DK. MADIBA: A web server toolkit for biological interpretation of Plasmodium and plant gene clusters. BMC Genomics. 2008;**9:**105

2. Salcedo-Amaya AM, van Driel MA, Alako BT, Trelle MB, van den Elzen AM, et al. Dynamic histone H3 epigenome marking during the intraerythrocytic cycle of Plasmodium falciparum. Proc Natl Acad Sci U S A. 2009;**106:**9655-9660.

Additional Table S1: Sequence statistics for ChIP-sequencing performed in this study of *Plasmodium falciparum*.

SN	Samples	Total Reads	Unique Reads	Source	GEO accession number
1	Input Rings	32,079,104	14,293,619	IISER, Pune	GSM1547552
2	Input Trophozoites	35,070,605	16,030,714	IISER, Pune	GSM1547553
3	Input Schizonts	34,445,594	16,953,112	IISER, Pune	GSM1547554
4	H3K4me3 Rings	18,907,588	09,544,802	IISER, Pune	GSM1547561
5	H3K4me3 Trophozoites	39,590,191	21,645,073	IISER, Pune	GSM1547562
6	H3K4me3 Schizonts	37,674,527	20,898,339	IISER, Pune	GSM1547563
7	H3K4me2 Rings	18,293,031	9,632,411	IISER, Pune	GSM1547558
8	H3K4me2 Trophozoites	39,765,501	22,445,724	IISER, Pune	GSM1547559
9	H3K4me2 Schizonts	37,584,468	21,567,161	IISER, Pune	GSM1547560
10	H3K4me1 Rings	53,036,091	15,622,070	IISER, Pune	GSM1547555
11	H3K4me1 Trophozoites	42,075,070	10,394,974	IISER, Pune	GSM1547556
12	H3K4me1 Schizonts	39,996,420	15,184,404	IISER, Pune	GSM1547557
13	H3K9ac Rings	29,409,217	14,883,877	IISER, Pune	GSM1547564
14	H3K9ac Trophozoites	33,134,046	14,472,407	IISER, Pune	GSM1547565
15	H3K9ac Schizonts	34,277,436	16,501,918	IISER, Pune	GSM1547566
16	H3K14ac Rings	28,807,980	15,114,365	IISER, Pune	GSM1547570
17	H3K14ac Trophozoites	33,240,504	14,939,580	IISER, Pune	GSM1547571
18	H3K14ac Schizonts	33,048,458	16,472,820	IISER, Pune	GSM1547572
19	H3K27ac Rings	43,794,654	1,763,346	IISER, Pune	GSM1547573
20	H3K27ac Trophozoites	59,494,138	2,646,663	IISER, Pune	GSM1547574
21	H3K27ac Schizonts	38,934,545	1,769,273	IISER, Pune	GSM1547575
22	H4ac Rings	44,267,508	26,762,078	IISER, Pune	GSM1547582
23	H4ac Trophozoites	38,640,318	16,589,677	IISER, Pune	GSM1547583
24	H4ac Schizonts	44,388,449	15,442,044	IISER, Pune	GSM1547584
25	H3K9me3 Rings	34,907,641	6,623,072	IISER, Pune	GSM1547567
26	H3K9me3 Trophozoites	32,881,747	7,010,653	IISER, Pune	GSM1547568

27	H3K9me3 Schizonts	36,583,216	4,503,280	IISER, Pune	GSM1547569
28	H3K79me3 Rings	29,948,048	10,059,303	IISER, Pune	GSM1547579
29	H3K79me3 Trophozoites	63,232,531	7,483,233	IISER, Pune	GSM1547580
30	H3K79me3 Schizonts	37,683,293	13,325,023	IISER, Pune	GSM1547581
31	H3K27me3 Rings	71,580,449	4,049,693	IISER, Pune	GSM1547576
32	H3K27me3 Trophozoites	30,559,535	1,668,129	IISER, Pune	GSM1547577
33	H3K27me3 Schizonts	42,957,936	1,755,895	IISER, Pune	GSM1547578
34	Histone H3 Rings		16,234,932	IISER, Pune	GSM1547585
35	Histone H3 Trophozoites		13,479,404	IISER, Pune	GSM1547586
36	Histone H3 Schizonts		14,165,066	IISER, Pune	GSM1547587

Additional Table S2: Sequence statistics for ChIP-sequencing and RNA sequencing from GEO data base for *Plasmodium falciparum*.

SN	Sample	Total Reads	Source	GEO accession number
1	H3K4me3 Rings	16,019,252	NHLBI, NIH	SRX278216
2	H3K36me2 Rings	09,975,379	NHLBI, NIH	SRX278212
3	H3K36me2 Schizonts	13,323,607	NHLBI, NIH	SRX278213
4	H3K36me3 Rings	10,963,984	NHLBI, NIH	SRX278214
5	H3K36me3 Schizonts	11,918,973	NHLBI, NIH	SRX278215
6	H3K9me3 Rings	11,945,913	NHLBI, NIH	SRX278218
7	H3K9me3 Schizonts	12,899,941	NHLBI, NIH	SRX278219
8	H4K20me3 Rings	12,040,010	NHLBI, NIH	SRX278220
9	H4K20me3 Schizonts	11,775,490	NHLBI, NIH	SRX278221
10	H2A.z Rings	5,500,000	NCMLS, Netherlands	SRX026758
11	H2A.z Trophozoites	5,500,000	NCMLS, Netherlands	SRX026760
12	H2A.z Schizonts	5,500,000	NCMLS, Netherlands	SRX026761
13	RNA seq 5hpi	13,617,055	NCMLS, Netherlands	SRX026750
14	RNA seq 10hpi	14,878,153	NCMLS, Netherlands	SRX026751
15	RNA seq 15hpi	13,312,805	NCMLS, Netherlands	SRX026752
13	RNA seq 20hpi	13,472,352	NCMLS, Netherlands	SRX026753
14	RNA seq 25hpi	15,449,937	NCMLS, Netherlands	SRX026754
15	RNA seq 30hpi	12,328,314	NCMLS, Netherlands	SRX026755
14	RNA seq 35hpi	19,286,750	NCMLS, Netherlands	SRX026756
15	RNA seq 40hpi	14,685,226	NCMLS, Netherlands	SRX026757

Additional Table S3: Sequence statistics and accession number of human histone modifications.

SN	Samples	Unique Reads	Source	GEO accession number
1	Input	03,997,695	BCH, Boston	GSM908072
2	H3K4me3	02,477,162	NHLBI, NIH	GSM317587
3	H3K4me2	10,535,411	BCH, Boston	GSM908036
4	H3K4me1	11,394,632	NHLBI, NIH	GSM317586
5	H3K9ac	14,048,450	BCH, Boston	GSM908049
6	H3K14ac	10,286,660	NCMLS, Netherlands	GSM566174
7	H3K27ac	04,881,487	BCH, Boston	GSM908050
8	H3K9me3	06,988,838	BCH, Boston	GSM908040
9	H3K79me3	10,396,173	NCMLS, Netherlands	GSM566173
10	H3K27me3	12,706,292	BCH, Boston	GSM908042
12	H2A.z	09,896,954	NHLBI, NIH	GSM317582
13	H3K36me3	14,481,568	NHLBI, NIH	GSM317585
14	H3K36me2	18,873,140	BCH, Boston	GSM908044
15	H4K20me3	05,938,007	RU, Netherlands	GSM749527

Additional Table S4. Correlation of histone modifications and transcription including and excluding virulence genes. Pearson correlation (r) was calculated between the median of gene expression and histone enrichment including and excluding virulence genes (first three subsets in Figure 3A, which are also enriched with H3K9me3). H3K36me2 shows negative correlation with expression (Pearson correlation -0.79) when the virulence genes are excluded.

Histone Modifications	Pearson correlation (all genes)	Pearson correlation (virulence genes excluded)
H3K4Me1	0.81	0.79
H3K4Me2	0.80	0.79
H3K4Me3	0.81	0.80
H3K9ac	0.88	0.87
H3K14ac	0.85	0.87
H3K36me2	0.24	-0.79
H3K36me3	0.69	0.70
H4ac	0.58	0.69
H4K20me3	0.61	0.68
H4K79me3	0.62	0.69

Additional Table S5. List of genes and their co-ordinates for each cluster is provided in a separate excel file.

Additional Table S6. Co-ordinates for transcription start sites (TSSs) and transcription termination sites (TTSs) for all the *Plasmodium falciparum* genes.

Additional Table S7: Primers used for ChIP-qPCR and PCR.

SN	Gene name	Primer	Used for
1	PF11_0468_Sense_RT_R	AACATGCTCATTTCTATTTTTGACA	RT-R
2	PF11_0468_Sense_PCR_F	TGTGCACATGGGAATTTCA	PCR-F
3	PF11_0468_Sense_PCR_R	CTCTTCAATAGCATCCTCTTCATT	PCR-R
4	PF11_0468_antiSense_RT_F	AACAAAAATGGGTTAGCTCATATAAA	RT-F
5	PF11_0468_antiSense_PCR_F	TTTGATGATATAGCACAAGGGAAG	PCR-F
6	PF11_0468_antiSense_PCR_R	TTTCCCTGTTGAACATTCCT	PCR-R
7	PF10_0287_Sense_RT_R	TCAGGAAGTGTTTGTATATTATTAATGC	RT-R
8	PF10_0287_Sense_PCR_F	GTAGACGTCGCAGTTCATGG	PCR-F
9	PF10_0287_Sense_PCR_R	TCAATATCGCTGTATAACTTTTCTTCA	PCR-R
10	PF10_0287_antiSense_RT_F	GGTTTAGTTTCCGCTGCAAT	RT-F
11	PF10_0287_antiSense_PCR_F	TTGGTTTCACTCACCTATGACA	PCR-F
12	PF10_0287_antiSense_PCR_R	ATCTGCTGGCCATACGTTTT	PCR-R
13	Pf11_0468	TGTGCACATGGGAATTTCA	ChIP-qPCR
14	Pf11_0468	CTCTTCAATAGCATCCTCTTCATT	ChIP-qPCR
15	Pf10_0287	CCATGAACTGCGACGTCTAC	ChIP-qPCR
16	Pf10_0287	AAAAATCCCTTAAAAAGATGAGTGA	ChIP-qPCR
17	PF13_0303	CAACCATCGTTCCTTGACCT	ChIP-qPCR
18	PF13_0303	GTAACCGTGCGTGTGCTTTA	ChIP-qPCR
19	PF13_0088	TTTTAAAGCATCTTCATTTGATGG	ChIP-qPCR
20	PF13_0088	AAATTGGATGAAATGTGATGATAA	ChIP-qPCR
21	PF11_0071	CACCTAACCCTTTTATATGACTGTG	ChIP-qPCR
22	PF11_0071	TTCGCCTTTGTGAAATAAAAAT	ChIP-qPCR
23	Control	AACGTTAAATTTTGAATCCGAGA	ChIP-qPCR
24	Control	AATCTCCGAGACCGGGAAT	ChIP-qPCR

Additional Table S8: Co-ordinates and primers used for the cloning of bidirectional promoters

Gene name	Co-ordinates	Forward primer	Reverse primer
PF08_0135	Chr8:1216102-1217144	GCGGCTAGCATTTCATAATGCAGCTGGGAAATA	GCGACCGGTTCGACTTGTTGGCTTGTTATAAAT
PF11_0210	Chr11:764608-765598	GCGGCTAGCTTTATAGAGATCCCGTTCAGTGAT	GCGACCGGTTTTTGATTTTCATCATTTCTCTT
